# *Stowaway* miniature inverted repeat transposable elements are important agents driving recent genomic diversity in wild and cultivated carrot

**DOI:** 10.1186/s13100-019-0190-3

**Published:** 2019-11-27

**Authors:** Alicja Macko-Podgórni, Katarzyna Stelmach, Kornelia Kwolek, Dariusz Grzebelus

**Affiliations:** Institute of Plant Biology and Biotechnology, Faculty of Biotechnology and Horticulture, University of Agriculture in Krakow, 31425 Krakow, Poland

**Keywords:** Transposable elements, Insertional polymorphism, TE-gene association, *Mariner*, *DcSto*, *Daucus carota*

## Abstract

**Background:**

Miniature inverted repeat transposable elements (MITEs) are small non-autonomous DNA transposons that are ubiquitous in plant genomes, and are mobilised by their autonomous relatives. *Stowaway* MITEs are derived from and mobilised by elements from the *mariner* superfamily. Those elements constitute a significant portion of the carrot genome; however the variation caused by *Daucus carota Stowaway* MITEs (*DcSto*s), their association with genes and their putative impact on genome evolution has not been comprehensively analysed.

**Results:**

Fourteen families of *Stowaway* elements *DcSto*s occupy about 0.5% of the carrot genome. We systematically analysed 31 genomes of wild and cultivated *Daucus carota*, yielding 18.5 thousand copies of these elements, showing remarkable insertion site polymorphism. *DcSto* element demography differed based on the origin of the host populations, and corresponded with the four major groups of *D. carota,* wild European, wild Asian, eastern cultivated and western cultivated. The *DcSto*s elements were associated with genes, and most frequently occurred in 5′ and 3′ untranslated regions (UTRs). Individual families differed in their propensity to reside in particular segments of genes. Most importantly, *DcSto* copies in the 2 kb regions up- and downstream of genes were more frequently associated with open reading frames encoding transcription factors, suggesting their possible functional impact. More than 1.5% of all *DcSto* insertion sites in different host genomes contained different copies in exactly the same position, indicating the existence of insertional hotspots. The *DcSto*7b family was much more polymorphic than the other families in cultivated carrot. A line of evidence pointed at its activity in the course of carrot domestication, and identified *Dcmar*1 as an active carrot *mariner* element and a possible source of the transposition machinery for *DcSto*7b.

**Conclusion:**

*Stowaway* MITEs have made a substantial contribution to the structural and functional variability of the carrot genome.

## Background

Transposable elements (TEs) are discrete segments of DNA capable of changing their genomic location in a process called transposition [1]. Based on the mechanism of transposition, TEs are divided into two classes, class I (retrotransposons), mobilised via an RNA intermediate, use a ‘copy and paste’ mechanism, while class II (DNA transposons) are mobilised by ‘cut and paste’ or ‘copy and paste’ mechanisms of DNA that do not require a reverse transcription step. In both classes, there are autonomous elements that possess enzyme-encoding genes required for mobilisation, non-autonomous elements which can still be mobilised by their autonomous counterparts, and inactive defective copies [2].

Miniature inverted-repeat transposable elements (MITEs) are small in size (< 800 base pairs, bp), usually AT-rich sequences with no coding capacity. They are mobilised by related autonomous class II trans-acting elements. Despite their small size, they may account for a significant portion of plant genomes, representing up to 10 and 13.8% for rice and mulberry, respectively [3, 4]. This extremely efficient proliferation of MITEs, as compared to their ancestral autonomous elements, might be caused by their higher affinity for transposase, resulting from lower *cis*-requirements for enzyme recognition and by the presence of subterminal and/or internal enhancers of nucleoprotein complex formation [5, 6]. Some MITEs families, such as *mPing* elements in rice, may preferentially target single-copy gene-rich regions [7], and escape the epigenetic control system because MITE-derived trans-acting siRNAs do not share sequence similarity with the coding region of the source of the transposase [8]. All these features, coupled with their small size, make them abundant in plant genomes, and frequently present in the vicinity of genes.

Currently, the pivotal role of TEs in the evolution of plant genomes is becoming more widely recognised. Mobilisation of TEs leads to structural variations that contribute to the genomic diversity of their host, some of which can be adaptive. Among other effects, the role of TE insertional hotspots in the formation of biosynthetic gene clusters was proposed, based on the analysis of genes of the terpene biosynthesis pathway in eudicots [9]. TE insertions can impact gene expression in many ways. By insertion upstream, within, or downstream of coding regions, TEs may provide new regulatory features that can affect gene expression [10]. In addition, RNA-directed methylation (RdDM), which has a role in repetitive DNA control and defense against viruses, can lead to epigenetic changes upon TE insertion that may produce epialleles for adjacent genes [11]. In crops, such modifications can affect agronomically important traits, such as observed with flowering time variation due to MITE insertions into the quantitative trait locus *Vegetative to generative transition 1 (Vgt1*) [12].

Therefore, one of the main challenges of crop genomics is to critically evaluate the extent of species-wide TE-associated structural variation (TEASV), in order to better understand the dynamics of genome evolution. Several bioinformatics tools have been developed that allow for the identification of TEASV from resequencing data generated by next-generation sequencing (NGS) (reviewed in [13]). However, only a few genome-wide comparative analyses of TEASV have been published, almost exclusively for autogamous species. Moreover, most of these were focused on the global TE landscape, and thus they were biased towards the most numerous TE families.

TE-derived variants are also sources of functional variation, as shown by TE variants linked to changes in DNA methylation in *Arabidopsis thaliana* [14] and flowering traits in maize [15, 16]. A study associating TE-derived variants with phenotypic traits related to maize adaptation to a temperate climate revealed more candidate genes than analysis using single nucleotide polymorphisms [16]. This suggests that TEs may be able to quickly enhance host adaptability under adverse environmental conditions. An adaptive role for TEs has also been suggested for *A. thaliana* [17] and *Capsella rubella* [18].

To date, only a few reports have focused on the global analysis of MITEs at the species level. Mining of MITEs in 19 *A. thaliana* ecotypes yielded a total of 2406 copies grouped into 212 families [19]. In another report, 20 MITE families were annotated in *B. rapa, B. oleracea*, and *A. thaliana*. Of these, only four were present in *A. thaliana*, indicating that amplification and diversification of most *Brassica* MITE families took place after divergence of the *Arabidopsis* and *Brassica* lineages. Moreover, some MITE families were significantly enriched in *B. rapa* and *B. oleracea*; therefore, they were likely activated after divergence of those species [20]. Rice *mJing* elements were frequently inserted into introns (16.67%) and into the 2 kb regions flanking genes (45.83%) [21]. Rice accessions differ dramatically in terms of *mJing* copy number, ranging from 18 to 150 in japonica and African cultivated rice, respectively. This suggested multiple amplification bursts, which most likely occurred before the amplification burst of *mPing*, another well-characterised active MITE in rice [21]. Some rice lines showed a sharp increase of *mPing* copies, from less than 10 copies in indica to 1000 in a temperate japonica cultivar Gimbozu EG4 [22]. Association of MITE insertions with coding regions was also shown for 18 wheat *Stowaway* element families, with 5.1% of more than 19,000 MITEs being transcribed, and 52–63% insertion sites being located within 100 bp of genes [23, 24]. A comparative analysis revealed specific proliferation of two MITE families in the A genome and one in the B genome, suggesting their possible impact on genome diversification during speciation [23].

Carrot (*Daucus carota*) is a diploid species with 2n = 2x = 18, and a relatively small genome of 473 Mb [25]. It is an allogamous species, suffering from inbreeding depression. Cultivated carrot is a biennial root vegetable and the most economically important species of the *Apiaceae* family, and is grown around the world in temperate and subtropical regions [26]. *D. carota* has been domesticated relatively recently, about 1100 years ago. Wild carrot is widespread in temperate regions of the world. While the Mediterranean basin is considered the centre of biodiversity for *Daucus* spp. [27], Central Asia has been identified as the place of origin of domesticated carrots [25, 28]. The species has four major structural groups: European wild *D. carota*, which show remarkable morphological diversity and are grouped into several subspecies, referred to as *D. carota* complex; Asian wild *D. carota* subsp. *carota*; eastern cultivated carrots, which are mostly primitive landraces, often producing yellow or purple storage roots; and western cultivated carrots, which include advanced orange cultivars. Cultivated and wild carrots can easily hybridise, and a considerable amount of genetic variation is exhibited both between and within the groups, with no apparent signature of a domestication bottleneck [28].

The carrot reference genome assembly of a double haploid plant (DH1) has been published recently [25]. The repetitive fraction constituted 46% of this carrot genome. DNA transposons comprised 13.6% of the genome and 30% of the total repetitive DNA. Approximately 2.3% of the assembled portion of the genome was attributed to MITEs, of which *Stowaway*-like elements constituted around 0.5% [25]. Carrot *Stowaway*-like MITEs (*DcSto*s) had previously been reported to be abundant and highly polymorphic [25, 29]. In this current study, we used 14 *DcSto* families for a systematic genome-wide analysis of TEASVs in 31 resequenced genomes from cultivated and wild carrot accessions. The accessions were representative of the four structural groups of *D. carota*, as described above. *DcSto* insertions were comprehensively annotated and their chromosomal distribution was analysed. In addition, we identified a *DcSto* family likely active in cultivated carrot.

## Results

### Distribution of *DcSto* elements in *D. carota*

In total, 18,518 *DcSto* insertion sites were identified across 31 genomes of *D. carota* (Table 1 and Additional file 1). Although the coverage of the resequenced genomes ranged from approximately 10× to 40× (50.8–225.3 million reads), this did not affect the sensitivity of insertion detection, as no correlation between the number of reads and the number of identified insertion sites was observed (Spearman rank correlation rho = − 0.12, *p* = 0.52; Additional file 2: Figure S1). In addition, the reference genome was similarly covered by reads from the resequenced accessions, spanning from 93.8 to 96.8% of the assembly [25], with 89.88 to 97.52% of total reads mapped (Additional file 2: Table S1). This indicated that the resequencing data did not show any significant bias, and that they were robust enough to be used for comparative analysis.

**Table 1:** Abundance and distribution of the 14 *DcSto* families in *D. carota*

*DcSto*		Number of *DcSto* insertion sites							
	Total	UIS^a^	PrC^b^	2 kb upstream	5’UTR^c^	cds^d^	intron	3’UTR	2 kb downstream
*DcSto1*	1685	1145	12.70	468	40	4	388	32	265
*DcSto2*	2594	1739	12.20	763	92	10	450	45	441
*DcSto3*	821	489	10.39	238	28	4	166	24	145
*DcSto4*	315	153	6.88	70	9	1	87	4	62
*DcSto5*	1385	916	11.16	419	53	0	204	27	260
*DcSto6*	3633	2512	13.23	932	87	8	903	86	702
*DcSto7a*	1484	983	12.10	456	38	9	296	37	266
*DcSto7b*	2887	2284	17.50	972	168	17	428	91	527
*DcSto7c*	256	143	9.85	70	9	0	48	7	55
*DcSto8*	857	637	12.16	233	35	6	210	13	145
*DcSto9*	266	140	9.82	73	11	1	57	7	39
*DcSto10*	301	184	9.87	85	11	0	34	11	49
*DcSto11*	155	72	6.33	41	4	1	23	3	35
*DcSto12*	1587	1068	11.18	393	60	6	323	52	336
PIS^e^	292		–	83	10	0	30	3	58
Total/average	18,518	12,464	11.10	5296	655	67	3647	442	3385

^a^*UIS* Unique insertion sites, ^b^*PrC* Proliferation coefficient (total number of insertions/average number of insertions per plant), ^c^*UTR* Untranslated region, ^d^*cds* coding sequence, ^e^*PIS* Parallel insertion sites

We further validated the results of in silico predictions for 39 randomly chosen *DcSto* insertion sites, using intron length polymorphism (DcS-ILP) genotyping, as described by Stelmach et al. [30]. For 16 sites, the results of DcS-ILP genotyping fully supported RelocaTE predictions. At 12 sites, other allelic variants were occasionally present, differing in size from the predicted *DcSto* insertion or the empty site, while for the remaining 11 sites no scorable polymerase chain reaction (PCR) products were produced. For the 28 sites yielding unambiguous PCR products, more than 96% RelocaTE predictions for the accession and site combinations were confirmed by the DcS-ILP assay (Table 2). This demonstrated that the applied in silico strategy reliably identified *DcSto* insertions.

**Table 2:** Verification of in silico results for 28 *DcSto* insertion sites in the carrot genome by DcS-ILP

Comparison of in silico / DcS-ILP results	Accession / insertion site combinations	
	number	%
empty / homozygous empty	541	64.4%
occupied / homozygous occupied	128	15.2%
occupied / heterozygous (empty + occupied)	94	11.2%
empty / homozygous variant of different size (‘empty’ for *DcSto*)	30	3.6%
empty / heterozygous (empty + variant of different size)	13	1.6%
occupied / heterozygous (occupied + variant of different size)	1	0.1%
Total correct calls	807	96.1%
empty / heterozygous (empty + occupied)	11	1.3%
empty / homozygous occupied	8	1.0%
occupied / homozygous empty	1	0.1%
Total incorrect calls	20	2.4%
No amplification	13	1.5%
Total	840	100%

All *DcSto* families had similar densities in all the nine carrot chromosomes (Additional file 2: Figure S2); however, they differed in terms of their copy number, from 155 copies for *DcSto*11 to 3633 copies for *DcSto*6 (Table 1). The differences likely reflected their ability to proliferate once integrated in the genomes of *D. carota*. The proliferation coefficient (PrC), i.e., the proportion of the total number of insertions per family divided by the average number of insertions per genome, ranged from 6.33 for *DcSto*11 to 17.50 for *DcSto*7b (Table 1). PrC values correlated with the intra-family similarity (Additional file 2: Figure S3), indicating that PrC was a good measure of the recent expansion of particular *DcSto* families.

### Insertional polymorphism of *DcSto*s

Among the 18,518 insertion sites, only two of them harboured the same element insertion in all 31 genomes of *D. carota* (Additional file 2: Table S2), while 22 insertions (0.12%) were present in all genomes of cultivated carrots (Additional file 2: Table S3). We observed a high proportion of insertions in only one of the 31 *D. carota* genomes (66.2%; Additional file 2: Table S4), which we subsequently referred to as unique insertion sites (UIS). It was important to note that most UIS were likely not ‘unique’ in absolute terms, but only in relation to the collection of 31 plants of different origin investigated in this current study. Thus, the majority of them represented *DcSto* insertions occurring less frequently, but likely still shared among populations of *D. carota*. In general, the number of UIS in the cultivated carrot accessions was relatively more uniform than in those representing the wild carrots. In addition, the wild carrots, especially those of European origin, had a higher proportion of UIS per genome, as compared to the cultivated carrots.

### *DcSto*s and the structure of genetic diversity for *D. carota*

**Fig. 1:**
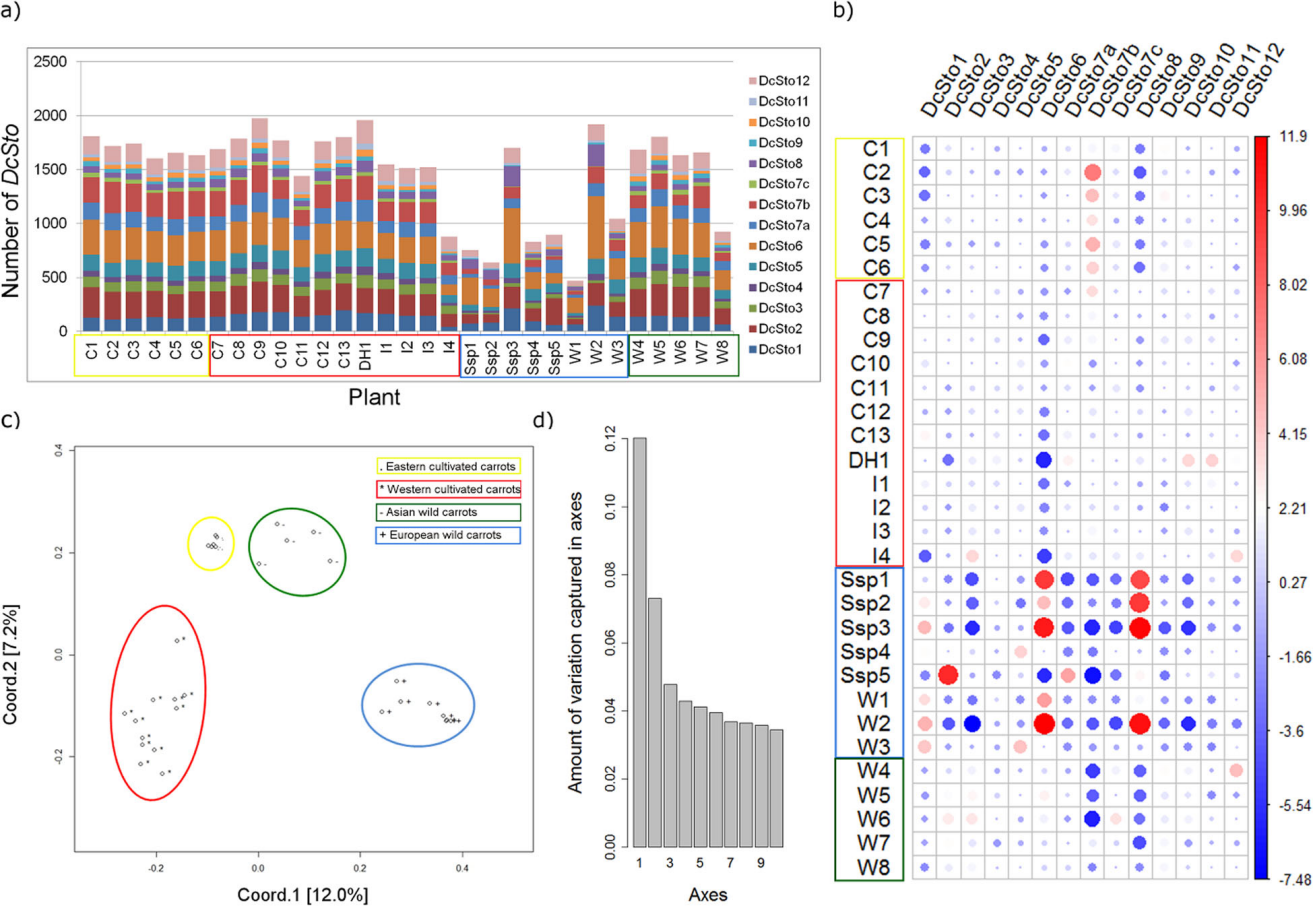
Distribution of *DcSto* copies among the 31 genomes of *D. carota*. **a** Contribution of families to the total number of *DcSto* insertion sites. **b** Relative abundance of *DcSto* families in 31 *D. carota* genomes (*p* = 2.2e-16). Colour scale reflects deviations from the average value. Circle size is proportional to the contribution of each test to the total Pearson chi-squared score. **c** Principal coordinate analysis (PCoA) plot based on insertion polymorphisms of the 14 *DcSto* families among the 31 *D. carota* accessions. **d** The variation explained by the first 10 axes of the PCoA. Western and eastern cultivated carrots, and European and Asian wild carrots are marked with red, yellow, blue, and green circles, respectively. Genetic distances were calculated using the Jaccard coefficient

The average number of *DcSto* insertion sites per *D. carota* genome was greater than 1400, ranging from 468 to 1978 (Fig. 1a). In the cultivated carrots, the number of *DcSto* copies was slightly higher (1655; ranging from 876 to 1978) than in the wild carrots (1226; ranging from 468 to 1920). As there was no apparent bias resulting from different coverage of the resequenced genomes, the four-fold difference was likely a biological phenomenon. Accessions of cultivated carrots (C1–C13) and inbreds (I1–I3) shared roughly similar numbers of *DcSto* copies. A purple carrot inbred line, B7262 (I4), was the only exception, as it carried far fewer *DcSto*s copies. More pronounced differences were observed in the wild *D. carota* gene pool. A group of accessions mostly originating from the West Mediterranean (the centre of biodiversity for *D. carota*) carried less than the average number of *DcSto* copies, while *DcSto* abundance in most wild Asian accessions (W4-W7) was similar to that of the cultivated accessions. This suggested variable dynamics of *DcSto*s elements in geographically separated wild populations.

*DcSto* families differed in terms of their contribution to the total copy number in individual genomes (*p*-value = 2.2e-16). The differences in *DcSto* distribution reflected the classification of the investigated accessions into four major groups, European wild (Ssp1-Ssp5 and W1-W3), Asian wild (W4-W8), eastern cultivated (C1-C6), and western cultivated (C7-C13 and I1-I4; Fig. 1b), in agreement with the previously reported population structure of *D. carota* [25, 28]. This was further confirmed by the genetic diversity structure inferred from global *DcSto* insertion polymorphisms. The four major groups were clearly distinguishable as non-overlapping clusters (Fig. 1c and d).

The eastern cultivated carrots were characterised by fewer *DcSto*1 and *DcSto*8 copies, while they carried more *DcSto*7b copies. Different proportions were observed in western cultivated carrots, which had slightly less *DcSto*6 copies. Within the Asian wild carrots, as in the case of the eastern cultivated carrots, *DcSto*1 and *DcSto*8 families were less numerous. By contrast, the eastern cultivated and the Asian wild accessions largely differed in the number of *Dcsto*7b copies, which were overrepresented in the former and underrepresented in the latter. Generally, European wild carrots were the most diverse in terms of *DcSto* distribution. Within this group, accessions Ssp1, Ssp2, Ssp3, and W2 had more *DcSto*6, *DcSto*8, and *DcSto*1 copies, while Ssp5 (*D. carota* subsp. *capillifolius*) was characterised by more *DcSto*2 and *DcSto*7a copies (Fig. 1b).

### Localisation of *DcSto* copies in relation to genes was non-random and family-specific

**Fig. 2:**
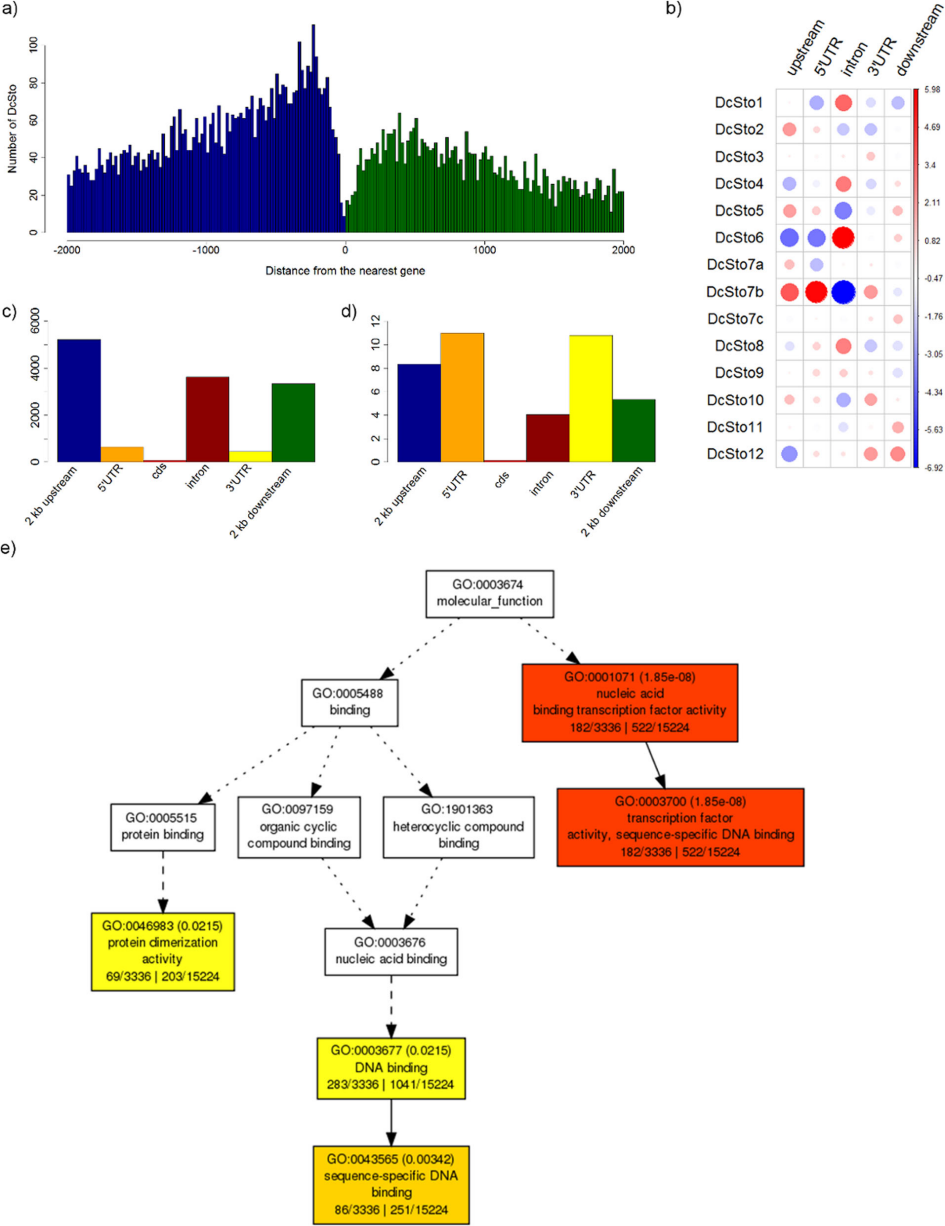
The number, distribution, and functional annotation of *DcSto* insertion sites within genic regions*.* **a** The number of *DcSto* insertions within 2 kb of the nearest gene in windows of 20 bp, with the up- and downstream regions being coloured blue and green, respectively. **b** The number of *DcSto* insertions in up- and downstream regions, exons (cds), introns and UTRs. **c** The number of *DcSto* insertions per 100 kb (standardised to the cumulative length of each region). **d** Differences in the distribution of *DcSto* families within genic regions (*p* = 5e-4), with cds regions not being included in the analysis. Colour scale reflects deviation from the average value. The size of circles is proportional to the contribution of each test to the total Pearson chi-squared score, and the number inside each cell is the Pearson’s residual. **e** Singular enrichment analysis (SEA) of all *DcSto*-associated genes, using AgriGO to define molecular functions

More than 73% of *DcSto* insertions were localised in genic regions, defined as insertions in genes and sequences 2 kb up- or downstream (Table 1). In absolute numbers, *DcSto*s elements were most frequently present in 2 kb upstream regions (28.4%), introns (21.7%), and 2 kb downstream regions (18.3%), while they were virtually absent in exons (Table 1, Fig. 2a and c). The number of insertion sites adjusted for the cumulative length of each defined genic region segment indicated enrichment of *DcSto* insertions in 5′ and 3′ untranslated regions (UTRs), with about 11 insertions per 100 kb of UTR, as compared to 4.5 insertions per 100 kb of introns (Fig. 2d). *DcSto* families differed with respect to their distribution within the genic region (Pearson’s chi-squared test, *p* = 5e-04). *DcSto*7b showed a higher than average proportion of insertions upstream of genes and within 5’UTRs, and a lower than average proportion of insertions within introns. By contrast, the most numerous family, *DcSto6*, showed the opposite pattern, being overrepresented within introns and underrepresented upstream of genes and within 5’UTRs (Fig. 2b).

Gene ontology (GO) enrichment analysis revealed that *DcSto* copies inserted in upstream or downstream regions of genes were significantly associated with those involved in the regulation of transcription (biological process, *p*-value = 4.06e-13; Additional file 2: Figure S4) and transcription factor activity (molecular function, *p*-value = 1.85e-08; Fig. 2e). By contrast, *DcSto*s elements inserted in introns did not show an association with any particular GO term, except for marginally significant family-specific signals not related to transcription regulation. For UTRs, the number of *DcSto* insertions were too low to find reliable associations, except for *DcSto*7b insertions in 5’UTR regions, which were significantly associated with genes encoding transcription factors (Additional file 2: Table S5).

### *DcSto* insertion hotspots

Within all identified insertion sites, 292 (1.6%) were parallel insertion sites (PIS), i.e., insertion sites of different *DcSto*s into precisely the same genomic position. Within PIS, 95% harboured insertions of *DcSto* copies from two different families, while the remaining 5% carried alternative insertions of three or more different copies. More than 63% of PIS were localised in the vicinity of genes or within the body of genes (Additional file 3).

**Fig. 3:**
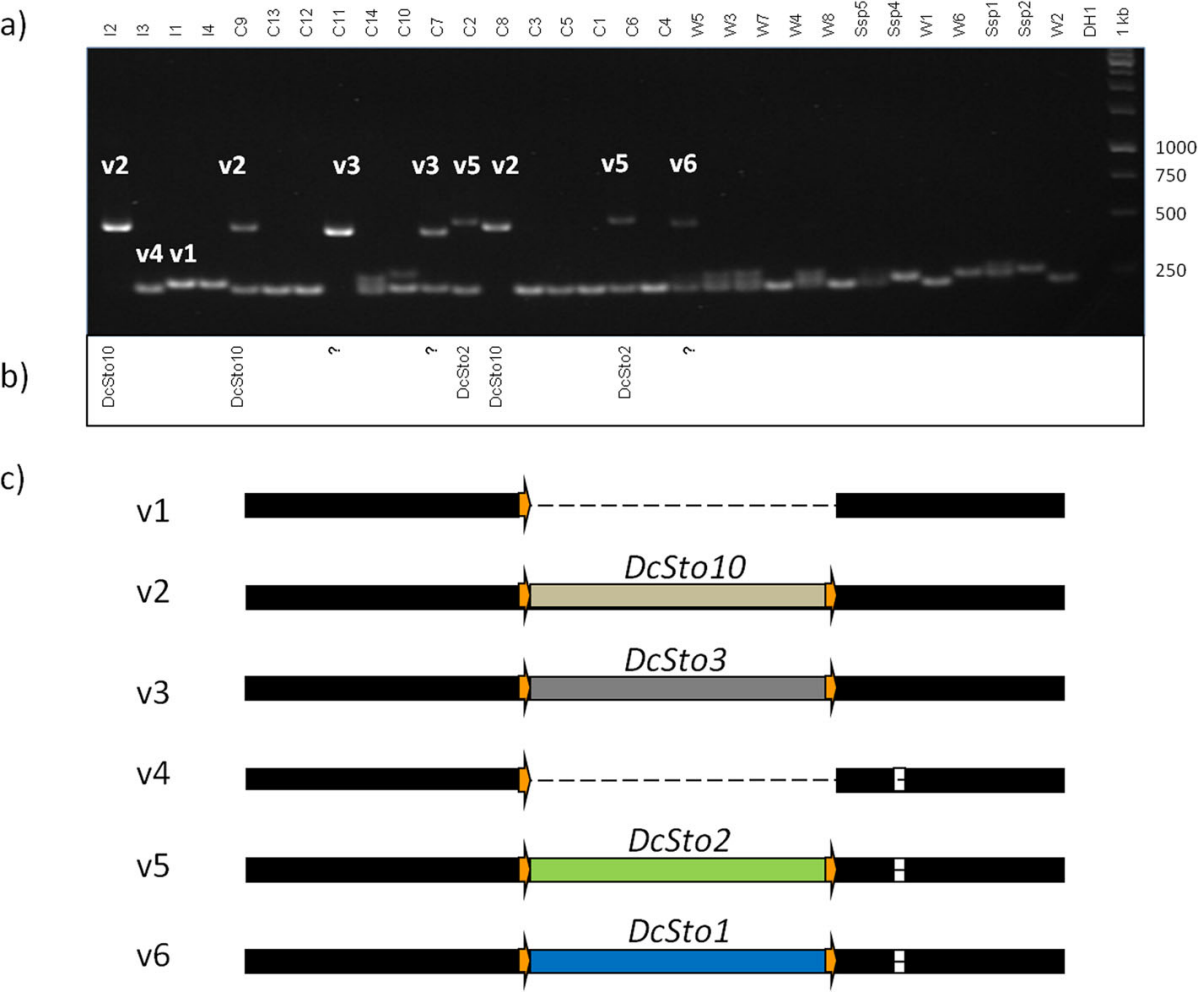
Verification of parallel insertions (PIS) at the DcS-MIS309 site. **a** PCR amplification profiles for variants (v1 to v6) labeled according to the schematic representation in (**c**). **b** Insertions identified by RelocaTE analysis. **c** Schematic representation of all identified insertion variants. White boxes show insertions and deletions (InDels) in the flanking region. The target site (TS) is represented by an orange arrow

To validate these in silico results, 11 PIS regions were PCR-amplified in the 30 resequenced accessions, and in the DH1 line as a reference, and the resulting amplicons were sequenced by the Sanger method. Presence of the in silico predicted PIS was confirmed in all instances (Fig. 3). Amplicons longer than the expected ‘empty’ variant were present in some accessions that were qualified as ‘empty’ by RelocaTE analysis. They carried additional rearrangements, e.g., an insertion of an unrecognised *Stowaway*-like MITE with terminal inverted repeat (TIR) sequences differing from the *DcSto* consensus or another unidentified insertion (Additional file 2: Figure S5), other *DcSto*s, or solo long terminal repeats (LTRs) in nearby positions within the amplicon (Additional file 2: Figure S6). PCR fragments shorter than the expected ‘empty’ fragment might represent deletion footprints created upon excision of a *DcSto* copy (Additional file 2: Figure S7). In addition, almost all PIS identified in silico as heterozygotes for particular individuals (each variant carrying a different *DcSto* copy) were positively verified by nucleotide sequencing (Additional file 2: Figure S8–S12). In silico identification of MITE insertion sites might be expected to be less reliable for PIS, as observed for the DcS-MIS309 site, where RelocaTE analysis failed to identify insertions in three plants, as revealed by the PCR screen (Fig. 3). Nevertheless, the combined results of in silico prediction and PCR verification suggested that insertions of different *DcSto*s elements and other MITEs into exactly the same genomic sites were quite common.

The co-occurrence of copies from two different *DcSto* families in PIS was positively correlated (*p* = 7.03e-12) with the cumulative number of all insertion sites of those families, and was negatively correlated (*p* = 8.80e-03) with the genetic distance between terminal inverted repeats (TIRs) of those families (Additional file 2: Figure S13, Additional file 2: Table S6). Thus, families with more copies were more frequent in PIS; however, *DcSto*s elements carrying more similar TIRs were also relatively more frequently inserted into the same site.

### *DcSto7*b elements have been active in the course of carrot domestication

**Fig. 4:**
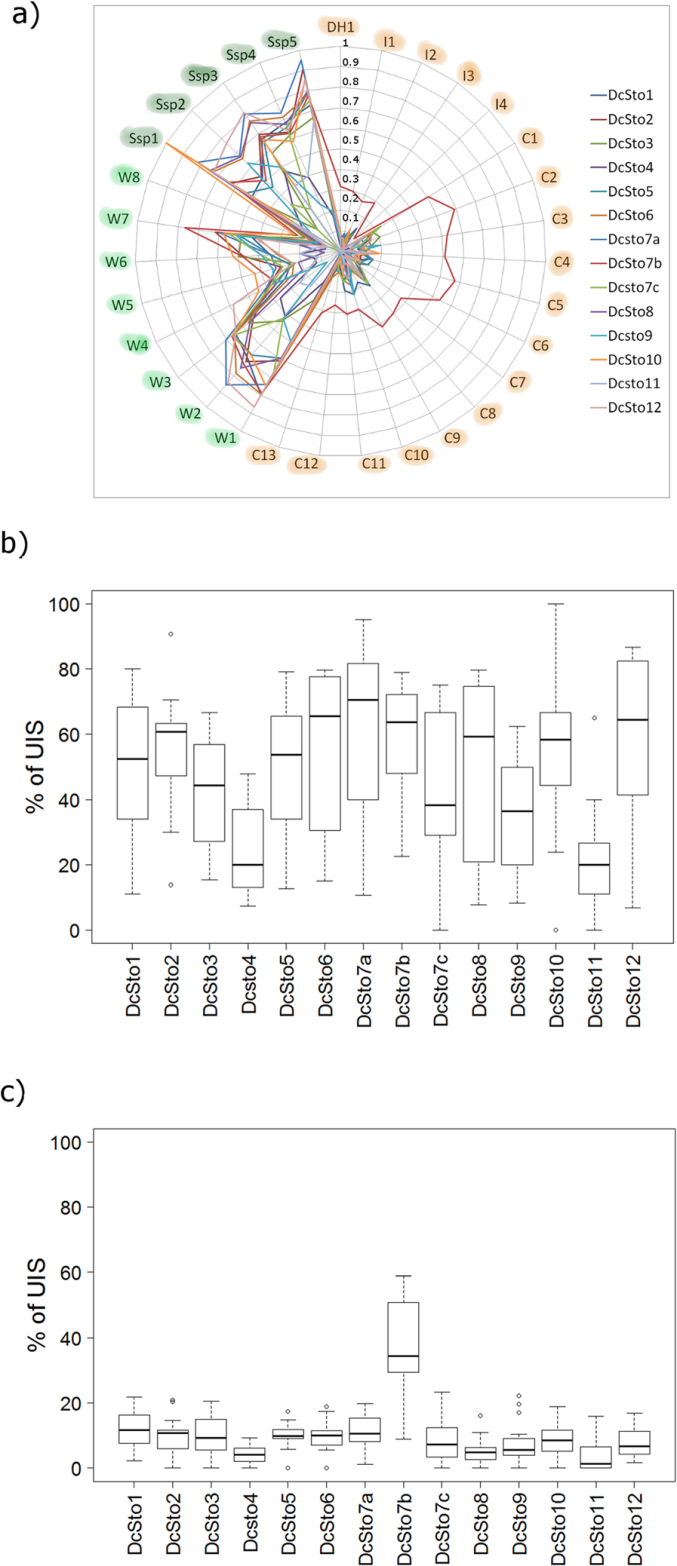
Distribution of unique insertion sites (UIS). **a** Proportion of unique insertion sites (UIS) to all insertion sites for the 14 *DcSto* families in 31 *D. carota* accessions, **b** in the cultivated, and **c** the wild *D. carota* accessions. Percent UIS was calculated as a proportion of the number of UIS to the total number of insertions for each *DcSto* family. The wild and the cultivated accessions are highlighted in green and orange, respectively

In cultivated carrots (both eastern and western), UIS of elements belonging to the *DcSto7*b family were exceptionally frequent (Fig. 4), accounting for an average of 38% (range 9–59%) of all insertions produced by the family. By contrast, UIS attributed to other *DcSto* families in the cultivated carrots ranged from 0 to 23%, with the average of 8% (Fig. 4b and c). In the reference genome (DH1), the *DcSto*7b family was characterised by the highest within-family similarity (96%) and a unimodal distribution of pairwise distances [25], suggesting a very recent burst in its activity. Combined with the present evidence, including (1) the high proportion of UIS in the genomes of cultivated accessions, (2) the highest PrC value, and (3) the unique pattern of insertion in relation to genes, as described above, it was likely that *DcSto7*b elements had been mobile in the cultivated carrot gene pool in the course of domestication.

### *Dcmar*1 might provide the transposition machinery for *DcSto7*b

The evidence for recent mobilisation of *DcSto*s elements described above prompted us to search for autonomous elements that could have been involved in the process. Eleven copies of *mariner*-like elements were found in the carrot DH1 genome (Additional file 2: Table S7), ranging from 1922 bp (*Dcmar*9) to 4940 bp (*Dcmar*6) and carrying 24- to 32-nt-long TIRs. One mismatch between the 5′ and 3′ TIRs was present in *Dcmar*1, *Dcmar*5, and *Dcmar*10, while TIRs of the remaining elements carried more mismatches (Additional file 2: Table S7). The C-terminal part of the predicted transposases of eight *Dcmar*s had a complete DD39D motif, characteristic of *mariner* elements. Three elements lacking the conserved region of the MLE domain (*Dcmar*9, *Dcmar*10 and *Dcmar*11) were classified as internally truncated and were not further considered.

The first two aspartic acids of the DD39D motif were predicted to be Mg^2+^ binding sites for all eight *Dcmar*s elements, while the helix-turn-helix (HTH) DNA binding motif was predicted for six of them, with at least a 90% probability (Additional file 2: Table S7). However, all features required for *mariner* transposition, as defined by Claeys Bouuaert and Chalmers [31], were only found with *Dcmar*1, a 4353 bp-long element inserted in chromosome 8 (position 25,189,375–25,193,731 in the reference genome DH1).

We investigated the transcriptional status of *Dcmar*s elements, using RNAseq reads of DH1 [25]. The *Dcmar*1 transposase was expressed in four of 20 tissues, callus, whole opened flowers (2 cm umbels at anthesis), bracts (2 cm umbels), and flower buds, while no transcripts attributed to other *Dcmar*s elements were found.

**Fig. 5:**
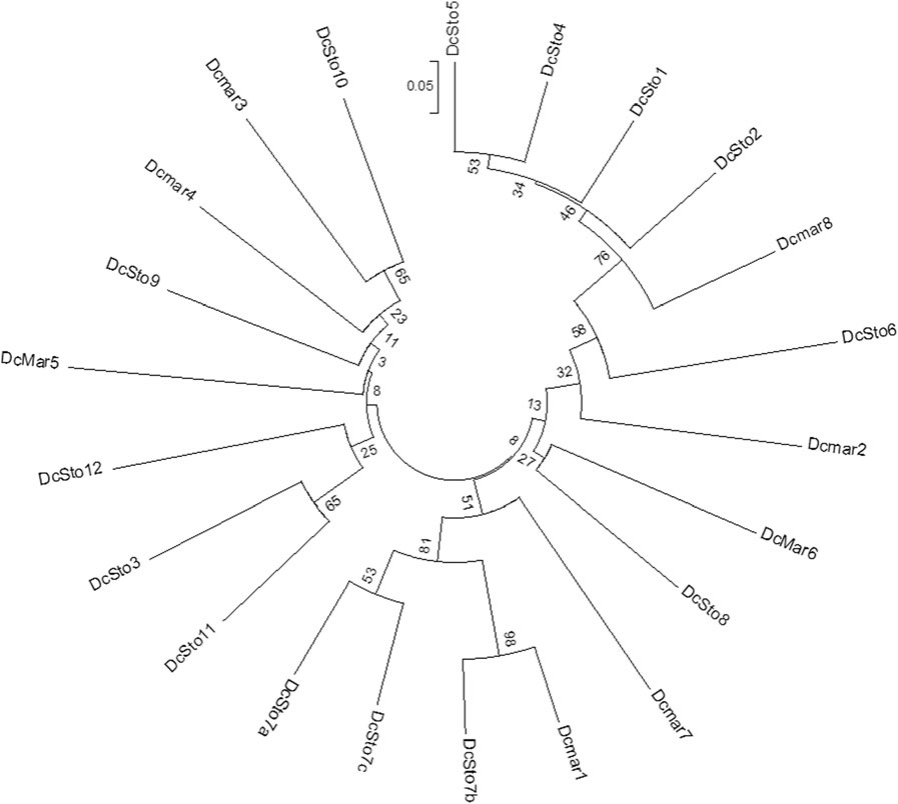
Neighbour joining tree showing evolutionary relationships of carrot *mariner*-like elements (*Dcmar*s) and *Stowaway* MITEs (*DcSto*s). The tree was generated based on the alignment of 100 nt-long 5′ and 3′ terminal sequences of elements. Numbers show bootstrap values

*Dcmar*1 and *DcSto*7b elements were the most similar with respect to their 100 nucleotide (nt) terminal sequences (Fig. 5). Both families shared 31 nt-long TIRs (5′ CTC CCT CCG TCC CTW TTT ATC TGT CCA HTT T 3′). Interestingly, most accessions harbouring a copy of *Dcmar*1 carried more *DcSto*7b copies, as compared to those lacking the autonomous element (Additional file 2: Figure S14a). However, not all accessions carrying *Dcmar*1 elements showed a *DcSto*7b copy number increase, indicating that the presence of *Dcmar*1 elements were essential; however, their activity likely depended on other factors, e.g., chromosomal position of the autonomous element. Therefore, the combined structural, transcriptomic and phylogenetic evidence suggested that *Dcmar*1 elements might have provided the transposition machinery for *DcSto*7b elements, driving their recent mobilisation in the gene pool of cultivated carrot.

At least one copy of *Dcmar*1 was present in 14 of 19 genomes of cultivated carrots (74%), but only 4 of 12 wild carrot genomes (33%; Additional file 2: Figure S14b). However, PCR amplification of the region spanning the *Dcmar*1 insertion site in the reference genome DH1 revealed an absence of the element at that genomic location in all other *D. carota* genomes investigated in this study (Additional file 2: Figure S14c). In silico identification of the position of *Dcmar1* elements was largely consistent with results from PCR assays. For all plants but one, we identified at least one putative insertion site, at 17 different genomic locations. Of these, eight *Dcmar*1 insertion sites were associated with genes. In the case of the eastern cultivated accession C4 from Afghanistan, probably only one insertion site was present in the genome; however, due to the insertion of *Dcmar*1 into an intron of one version of paralogues with high similar sequences, we were not able to determine its precise position (Additional file 2: Figure S14d). Only in case of wild carrot accession W2 from Portugal was the PCR assay positive; however, the presence of a *Dcmar*1 copy was not confirmed by in silico analysis. The data suggested that *Dcmar*1 elements had been actively transposing.

## Discussion

Transposable elements have been recognised as major drivers of the evolution of eukaryotic genomes. They have been involved in the creation of structural and functional novelty, making them an essential and long-standing part of genomes [32]. In particular, MITEs and LTR retrotransposons, being ubiquitous in plants, have dynamically shaped genome structure and altered gene function in a variety of ways [33]. A number of recent reports have provided clues of functional interactions of MITEs with host genes [4, 34–41]. The rice genome has been used as a model for investigating MITE-host interactions, as despite its moderate size it harbours an exceptionally rich and diverse collection of almost 180,000 MITE copies divided into 338 families [42], including *mPing*, the most thoroughly studied currently active MITE family [7]. However, less than 15% of all full-length MITE insertions were polymorphic between subspp. *Indica* and *japonica* [42]. Being a dicot species, carrot provides an alternative plant host model to rice. Our results point at a much higher level of insertional polymorphism of carrot *Stowaway* MITEs, perhaps more similar to that of active *mPing* elements in rice [22]. This might be attributed to the recent or ongoing activity of some *DcSto* families, but also to the fact that contrary to rice, carrot is an allogamous species. De la Chaux et al. [43] reported a major reduction of TE abundance in autogamous *A. thaliana*, as compared to its close allogamous relative *A. lyrata*, consistent with such theoretical expectations [44].

### *DcSto* mining strategy

Fourteen *DcSto* families were selected as they were the most abundant in the carrot reference genome [25]. A global annotation of MITEs in carrot also revealed the presence of several other *Stowaway*-like MITE families that were usually less numerous (data not shown). It was possible that some of them acquired much higher copy numbers in the resequenced genomes, as suggested by up to four-fold differences in the global number of analysed *DcSto*s elements, as well as within-family differences.

The genome-wide comparative analysis yielded a catalogue of 18,518 structural variants caused by MITE copies belonging to 14 *DcSto* families across 31 *D. carota* accessions. Of these, less than 2000 copies were attributed to the reference genome DH1. Previously, Iorizzo et al. [25] identified around 4000 *DcSto* copies in the carrot genome. However, in this current study a more stringent approach was used for *DcSto* mining, which resulted in a generally lower number of catalogued insertion sites. Iorizzo et al. [25] used the web tool TIRfinder [45], which identifies all sequences that share common structural features, i.e., specified target site duplications (TSD) and TIRs in assembled sequences. All elements meeting these criteria, regardless of their genomic location, were reported. By contrast, RelocaTE analysis [46] was used in the current study. It retrieved insertion sites from raw sequencing reads based on a similarity search using stringent cut-off parameters, filtering out reads mapping to genomic regions comprising repetitive sequences. Therefore, elements too divergent from the TE family consensus and those inserted into repetitive regions or in the vicinity of structural variants were not reported. This was why in this current study only complete MITEs residing in unique genomic regions were mined, and consequently, the total number of *DcSto* elements obtained for the reference genome was lower than previously reported. Nevertheless, the same approach was systematically applied to all carrot accessions, and the results were comparable. In addition, the in silico mining results were extensively validated by PCR, with the reliability of the mapping tool RelocaTE being largely confirmed.

### *DcSto* distribution and the genetic diversity of *D. carota*

*DcSto* insertions were extremely polymorphic between cultivated and wild carrots, and within both groups. Global *DcSto* insertion polymorphism revealed a genetic diversity that mirrored previous reports using single nucleotide polymorphisms [25, 47]. It showed that informative *DcSto* insertional polymorphisms, i.e., those present in at least two genomes, allowed grouping of the accessions. The high rate of unique insertion sites observed might have resulted from insufficient sampling, especially for the wild *D. carota* group; however, it might also indicate current transpositional activity. This latter option was supported by the observation that copy numbers of particular *DcSto* families differed among accessions belonging to the same group. For example, the Portuguese accessions (members of the wild European group) were enriched in *DcSto*6 and *Dcsto*8 elements, while subsp. *capillifolius* carried more copies of the *DcSto*2 family. This might suggest amplification bursts of different MITE families in geographically separated populations of wild carrots. In addition, while both eastern cultivated carrots and eastern wild carrots had similarly low frequencies of *DcSto*1 and *DcSto*8, they differed with respect to the numbers of *DcSto*7b elements. The sharp increase in the number of *DcSto*7b copies in eastern cultivated carrots, as compared to the sister clade of Asian wild carrots, suggested the activity of these elements might have been significant during the early stages of domestication.

### *DcSto* insertions in the context of the carrot genome

*DcSto* copies showed similar distribution patterns across all carrot chromosomes typical for MITEs, i.e. depletion around centromeres and enrichment in genic regions. Previously, fluorescence in situ hybridisation had revealed *DcSto* signals along chromosome arms, and their absence at centromeres, telomeres, and nucleolar organiser regions [48]. Iorizzo et al. reported that *DcSto*s elements did not show any deviation from a random distribution across the carrot reference genome [25]. Even so, the current study indicated that individual *DcSto* families were characterised by contrasting distribution patterns in terms of their association with genes. Enrichment of *DcSto*7b copies was observed within the 2 kb region upstream of transcription start sites (TSSs) and in 5′ UTRs, and depletion in introns, while *DcSto*6 showed the opposite tendency. This might reflect a genuine preference for insertion of different families into specific sections of genic regions, or random insertions followed by selection acting on non-neutral insertions. The latter scenario would change frequencies of insertions observed in different genic segments, depending on the age of the insertions. *DcSto*7b copies are very similar and the family has been shown to have had a single very recent peak of activity [25]. By contrast, *DcSto*6 elements are an older family, which has likely experienced several peaks of activity [25]. If the selection hypothesis is true, it would imply selection has acted against *DcSto*6 insertions in sequences upstream of genes and in 5′ UTRs and/or retention of insertions in introns.

To date, comparative analyses of TE distribution has usually been generalised for larger groups. A global distribution of MITEs in *Citrus* resembled that reported in this current study for *DcSto*s elements in carrot [49]. However, analyses focusing on particular TE families have revealed specific patterns [4, 50]. Notably, in contrast to carrot *Stowaways*, mulberry *Tc1*/*mariner* superfamily MITEs were the only group that was not preferentially inserted near genes. Nevertheless, they had the highest ratio of the total number of transcribed MITEs to the total number of genes [4]. This supported our hypothesis that detailed analysis of individual families was essential for better understanding of the impact of TEs on the host genome.

### Prevalence of low frequency *DcSto* insertions

We observed a high level of low frequency *DcSto* insertions, with most of them referred to as UIS if present in only one of the 31 investigated genomes, a phenomenon also reported for other species [17, 51, 52]. As proposed by Uzunović et al., localisation of TEs in genic region may be limited due to negative selection [53]. On the other hand, the prevalence of UIS may result from an ongoing TE activity. For rice retrotransposon families, Carpentier et al. suggested that the presence of both low- and high-frequency insertion sites indicated continuous transposition, while high numbers of low-frequency insertions indicated their recent mobilisation [52]. In general, most carrot *DcSto* families produced higher proportions of UIS in wild carrots, as compared to the cultivated carrots. With the absence of any significant domestication bottleneck in carrots [25, 28], such a difference was not expected, unless the transpositional activity of *DcSto*s had been elevated in the wild genepool. Alternatively, one might speculate that TE insertional polymorphisms are a more sensitive indicator of a domestication bottleneck than SNPs, due to non-neutrality of some gene-associated insertions.

### *DcSto* insertion hotspots

This current study showed that more than 1.5% of all *DcSto* insertion sites were occupied by more than one *DcSto* element in exactly the same position in different genomes, which we named parallel insertion sites (PIS). The occurrence of different *Stowaway* MITE insertions in orthologous positions has been reported previously, e.g., it was studied for the β-amylase gene in Poaceae [54, 55]. However, it has never been addressed in the context of whole genomes. In this current study, we showed that it was a relatively frequent phenomenon and new insertions appeared fast enough to produce a series of insertion variants within the species. Notably, different copies at the same insertion site usually came from families sharing more similar terminal sequences. This might suggest that they utilised the same source of transposase, which resulted in parallel targeting to the same chromosomal positions. In carrots, 63% of all PIS were located within 2 kb of the nearest gene. As such, it will be important to reveal if these variants show functional variability in these genes. Recently, the importance of variation sources resulting in the occurrence of parallel mutations has been highlighted [56].

### *DcSto*s as a source of variation in genic regions

Carrot *DcSto*s elements, like other MITEs, were frequently associated with genes. The current study showed that 73% of all *DcSto* copies were inserted in the vicinity of genes, and particular *DcSto* families differed in their distribution within genic regions. A similar distribution of MITEs, enriched upstream of TSSs and depleted within the body of genes, was observed for *Stowaways* elements in potato [57] and *mPing* elements in rice [58]. Some 9738 carrot genes, including 61 tRNA genes, were associated with at least one *DcSto* element. On average, 3% of all annotated genes were associated with *DcSto*s elements in an individual carrot genome, ranging from 337 genes for accession W1 to 1490 genes for accession C9 (Additional file 2: Table S8). It was likely that these insertions were important for the fine-tuning of the expression of these associated genes [58]. Indeed, the non-random association of *DcSto* insertions with particular groups of genes, most notably transcription factors, indicated functional importance of these associations. However, none of the gene-associated *DcSto* insertions was fixed in *D. carota*. Nevertheless, they might provide a rich source of variability in the fine-tuning of certain regulatory networks and constitute a basis for selection. A more extensive sampling across carrot germplasm will be required to verify if some of these insertions show signatures of selection during domestication.

A recent genome-wide analysis of TEs showed that they were very important for rapid genome modifications, providing phenotypic variability important for adaptation. In *A. thaliana* ecotypes, genes carrying polymorphic TE insertions were enriched for defense and immune response functions important for adaptation to new ecological niches [17]. At least two genes with TE insertions were likely positively selected, contributing to the adaptation of that species. Similarly, TEs were involved in the rapid adaptation and the “genetic paradox of invasion” of *C. rubella*. In comparison to its outcrossing relative, *C. grandiflora*, *C. rubella* promoter regions were enriched in TE sequences [18]. Variability resulting from polymorphic insertion sites of *Stowaway* MITEs and an altered methylation status of surrounding sequences may impact adaptation to local environmental conditions, as reported for wild emmer wheat [59, 60]. It was likely that *DcSto*s, especially *DcSto*7b, had contributed to the phenotypic variation of cultivated carrots. Carrot has been domesticated relatively recently [61]; however, it shows a remarkable diversity of cultivar types and storage root traits [62]. It was tempting to speculate that at least some of the observed variability among carrot cultivars could have resulted from selection on variants resulting from the insertion of *DcSto* elements.

### *DcSto7*b elements were activated upon domestication

To date, only a few active MITEs have been described for which an accompanying autonomous class II element was proposed. These include *Stowaway* family *dTStu1* element in potato [63], and *Tourist* and *hAT*-related MITE families in rice [7, 21, 64–67]. The current study indicated that the *DcSto*7b family had been mobilised in the course of carrot domestication and might still be active in cultivated carrots. The high proportion of UIS of *DcSto*7b elements in cultivated carrots was notable in relation to the opposite trend for the remaining *DcSto* families. Several lines of evidence suggested very recent activity by *DcSto*7b elements, namely the highest PrC value (Table 1), more copies in the cultivated carrot accessions (Fig. 1b), and more UIS as compared to other *DcSto* families (Fig. 4). This was further supported by the highest intra-family similarity of individual copies of *DcSto*7b in the DH1 reference genome, as reported previously [25].

We hypothesised that *Dcmar*1, a related autonomous *Mariner*-like element, provided the transposition machinery for the mobilisation of *DcSto*7b elements. *Dcmar*1 was present only in a subset of the studied carrot accessions, which showed higher *DcSto*7b copy numbers, being more frequently present in genomes of cultivated carrots. The insertion site of *Dcmar*1 in the DH1 reference genome was unique, with the same position being empty in all the remaining 30 plants. Therefore, *Dcmar*1 itself, was likely a currently active element.

## Conclusions

This current study described the landscape of carrot *Stowaway* MITEs, providing insight into their importance in shaping the structural and functional variability of the carrot genome. Extreme insertional polymorphism of carrot *Stowaways* was identified, likely resulting from their recent mobilisation, as well as diversification from amplification bursts among carrot accessions. In particular, the *DcSto*7b family had likely been active in the course of domestication. Moreover, *DcSto* insertions were commonly present within genic regions, and were non-randomly associated with specific groups of genes, including those encoding transcription factors, with independent insertions of MITEs in the same genomic positions being relatively common events (comprising 1.6% of all insertion sites). Further analyses of carrot MITEs will be needed to understand the mechanisms responsible for their successful amplification and the extent of their functional impact on genes and on the phenotype of carrots.

## Methods

### Plant materials

To identify *DcSto* insertions, we used sequencing data from 31 resequenced genomes of *D. carota* (NCBI Sequence Read Archive, accession SRP062070, under umbrella project PRJNA285926; Additional file 2: Table S1), comprising 13 wild and 18 cultivated carrot accessions, along with the assembled carrot reference genome and its raw reads [25]. DNA from the 31 resequenced plants (excluding Ssp3 and including C14) was amplified using a REPLI-g Mini Kit (Qiagen), following the manufacturer’s protocol.

### In silico mining of *DcSto* insertions

Raw reads were pre-processed by removing low quality reads and trimming adapters using Trimmomatic version 0.35 [68], with parameters minqual = 28, minlen = 50, LEADING:28, TRAILING:28, SLIDINGWINDOW:10:28, and MINLEN:50, and quality was controlled using fastqc [69].

To identify insertion sites of the 14 *DcSto* families we used RelocaTE [46] with consensus sequences representing *DcSto* families [25]. RelocaTE allowed identification of TE insertions from unassembled short reads. In brief, short reads were aligned to a reference/consensus TE sequence, matching reads were trimmed to remove the TE sequence, and the remaining read fragments were aligned to the reference genome to identify the regions flanking the TE insertions [46]. The following RelocaTE parameters were use: -bm 12, −bt 11, −m 0.2 and -r 1. As the method included a mapping step, we first examined whether there were differences in the percentage of reads aligning to the reference genome. The mapping quality was evaluated with bwa-mem [70], using previously described parameters [71]. Next, files containing information about insertion sites for each *DcSto* family/genome combination were merged and converted into a binary matrix using a custom script, with absence and presence of a TE insertion being scored as 0 and 1, respectively.

Due to differences in genome coverage, we calculated correlation between the depth of coverage and the number of identified insertion sites. The Shapiro-Wilk’s normality test was performed, and the non-parametric correlation was tested using Spearman’s rank-based correlation, with the results were plotted in R using the ‘ggpubr’ package v.0.2 [72].

A binary matrix for the 31 accessions was used to calculate the number of *DcSto* insertion sites, UIS, i.e., those present in only a single accession, and the number of PIS, i.e., those with different copies of *DcSto* elements inserted in different genomes at exactly the same position. Genomic distribution of *DcSto* insertion sites and genes was plotted using the ‘ggplot2’ R package [73].

The presence of *DcSto* insertions in the context of genic regions, divided into five categories of 2 kb upstream sequences, 5’UTRs, coding sequence (cds), introns, 3’UTRs, and 2 kb downstream sequences, were determined based on the *National Center for Biotechnology Information* (NCBI) carrot genome annotation file GCF_001625215.1_ASM162521v1_genomic.gff, using BEDTools v.2.26.0 [74]. The same resource was used to calculate the total length of each of the five genic categories. Singular enrichment analysis (SEA) of the *DcSto*-associated genes was carried out using the Phytozome annotation file (Dcarota_388_v2.0.annotation_info.txt) and AgriGO v.2.0 [75], to define biological processes (BP), cellular components (CC) and molecular functions (MF).

All correlation tests were calculated and plotted using the ‘Corrplot’ R package [76]. Family distribution of *DcSto* insertion sites within the five genic categories was calculated based on a contingency table of data representing the number of occurrences of each *DcSto* family in defined segments, using the Pearson chi-squared test. Due to a low number of *DcSto*11 insertions, a simulated *p*-value based on 2000 replicates was used. Pearson residuals were calculated using a contingency table containing data representing the total number of insertion sites of each *DcSto* family in individual genomes. The matrix of Pearson’s correlation coefficients was calculated to test interconnection between the sum of copy numbers for families that were inserted into the same position (PIS), the number of their common occurrences in PIS, and the genetic distance between each pair of *DcSto* consensus sequences. Intra-family genetic distance was calculated for all copies representing each family identified in the DH1 genome, as reported by Iorrizo et al. [25].

The binary matrix for the 31 genomes was used to calculate the genetic distance based on the Jaccard coefficient, with the ‘vegan’ R package [77]. This was a conservative approach, where only the presence of a common insertion was considered informative. The values were used for principal coordinate analysis (PCoA) using the ‘ape’ package in R [78].

Finally, a gff3 file was prepared, where for each insertion the ‘start position’ referred to the second nucleotide (A) of the target site (TA), while the ‘end position’ referred to the first nucleotide of the *DcSto* element, in the case of insertions present in the reference genome DH1, or to the first nucleotide following the target site, in the case of insertions not mapped to DH1. For each insertion, a note containing information about its genomic position was given, as well as the LOC number of the adjacent gene, when the *DcSto* copy was inserted in a genic region (less than 2 kb from the gene). The ID field contained information about the *DcSto* family to which the copy was attributed, and comma separated codes of accessions carrying the insertion.

### Identification of autonomous elements

Autonomous *mariner*-like elements were mined from the DH1 reference genome assembly using TIRfinder [45], with tirMask: CTCCCTYYSKYMC, tsdMask: TA, tirSeqMismatches: 1, tsdSeqMismatches: 0, tirMaskMismatches: 0 and tsdMaskMismatches: 0. Coordinates and sequences of identified elements were manually inspected to remove redundant sequences. FGENESH [79], GENEID [80] and Augustus [81] gene prediction tools were used to identify coding regions in all mined TE sequences.

Predicted proteins in TEs were aligned with transposase sequences of known plant *mariner*-like elements, from *Ppmar*1 (NCBI accession no. HM581665), *Soymar*1 (NCBI accession no. AF078934) and *OSMAR*1 (Repbase accession no. AC135425), using ClustalW [82]. The presence of a highly conserved fragment of the *mariner* transposase starting from the first two aspartic acids of the DDD motif, previously used for phylogenetic analysis of plant *mariner-*like transposases [83], was manually inspected. Elements lacking the DDD motif were removed from further analysis. For the remaining proteins of putative autonomous elements, HTH motifs [84] and iron binding sites [85] were identified. The basic local alignment search tool (BLAST) was used to compare the corresponding mRNAs with carrot DH1 RNAseq short reads from 20 tissues (Sequence Read Archive SRP062159) [25].

Phylogenetic analysis of putative autonomous *mariner*-like and *DcSto*s elements was conducted with Mega v.6.06 software [86]. Evolutionary distances were computed based on 50 nt-long sequences of both TIRs using the p-distance method [87], and were used to calculate a neighbour joining tree [88]. Bootstrap values were obtained based on 1000 replicates.

To identify genomic positions of *Dcmar*1 elements in the resequenced genomes, cleaned Illumina reads were analysed by the TRACKPOSON method [52]. One cultivated carrot accession, I4, was not included in the analysis, as only forward reads were available. To avoid false positives from *DcSto* MITEs, TIRs were removed from the *Dcmar*1 query sequence prior to analysis, leaving only the internal portion of the sequence specific to the *Dcmar*1 element. In order to precisely determine genomic positions, sequences flanking *Dcmar*1 elements were reconstructed from unmapped paired reads, manually verified, and aligned with the DH1 carrot reference genome using BLAST analysis. The presence of *Dcmar*1 TIRs in the reconstructed sequences provided a confirmation of the results of in silico mining.

### Experimental verification of *DcSto* insertion sites identified by RelocaTE analysis

Thirty-nine *DcSto* insertion sites located in introns, and six sites characterised by parallel insertions, were selected for validation. For PCR, site-specific primers were as described by Stelmach et al. [30], or were designed de novo using Primer3 [89] (Additional file 2: Table S9). Reaction mixes contained about 20 ng REPLI-g-amplified genomic DNA, 1 mM forward and reverse primers, 0.25 mM dNTPs (Thermo Fisher Scientific), 0.5 U Taq DNA polymerase (Thermo Fisher Scientific), and 1x Taq buffer with MgCl_2_ (Thermo Fisher Scientific). Amplification took place at 94 °C for 1 min, followed by 30 cycles of 94 °C for 30 s, 56 °C/58 °C for 30 s, and 68 °C for 2 min, and finally 68 °C for 6 min. Products were separated by 1% agarose gel electrophoresis, and were purified with a GeneJET Gel extraction kit (Thermo Fisher Scientific), and cloned into pGEM-T (Promega). Cloned DNAs were extracted using the Wizard Plus SV Miniprep DNA Purification System (Promega) and sequenced by the Sanger method (Genomed SA, Poland). Nucleotide sequences were manually aligned using BioEdit [90].

The presence of *Dcmar*1 elements in *D. carota* accessions was verified using a pair of primers, DcMar1_499_F: 5′ GCC GAC ATA CGA ATC CTG TCA 3′ and DcMar1_499_R: 5′ TTG TGG CTT CCT TCT GCT GTA 3′, anchored in the DDD domain of the *Dcmar*1 element. The presence of *Dcmar*1 in the DH1 insertion site was screened across *D. carota* accessions with one of the above DDD-anchored primers in combination with a corresponding forward or reverse primer flanking the insertion (DcMar1_499_flank_F: 5′ TGT TCT TAG CAG CGG TAG CAC and DcMar1_499_flank_R: 5′ GTT GGT GTT TAC ACT GGA GGT TG 3′). As a positive control for the PCRs, a single-copy carrot genomic fragment was amplified with primers CULT-q-orf6-F 5′ CTT CTC GTA CAA CTG AGC C 3′ and CULT-q-orf6-R 5′ GCT TAG CAA GTA CAA GGG AA 3′ [71]. Fragments were amplified in 10 μl reactions containing 20 ng REPLI-g-amplified genomic DNA, 1 mM forward and reverse primer, 1 mM forward and reverse control primer, 0.25 mM dNTPs (Thermo Fisher Scientific), 0.5 U Taq DNA polymerase (Thermo Fisher Scientific), and 1x Taq buffer with MgCl_2_ (Thermo Fisher Scientific). Amplification took place at 94 °C for 1 min, followed by 30 cycles of 94 °C for 30 s, 56 °C for 30 s, and 68 °C for 2 min for the DDD test and 10 min for the DH1 site, and then 68 °C for the final elongation for 6 min for the DDD test and 20 min for the DH1 site.

## Supplementary information

**Additional file 1: ***DcSto* annotation: contains gff3 annotations of 18.5 K *DcSto* insertions identified in 31 carrot genomes.

**Additional file 2:** Supplementary Figures and Tables: contains supplementary figures and tables referenced in the main manuscripts.

**Additional file 3:** Supplementary Table: contains a list of gene-associated parallel insertion sites (PIS) and their functional annotations.

## Data Availability

Reads of 31 resequenced genomes of *D. carota* were downloaded from the NCBI Sequence Read Archive database under the project number: SRP062070. Carrot genome annotation files were downloaded from the NCBI database (GCF_001625215.1_ASM162521v1_genomic.gff) and the Phytozome database (Dcarota_388_v2.0.annotation_info.txt). DH1 transcriptome reads were downloaded from the NCBI Sequence Read Archive database, project number SRP062159. All data generated during this study are included in the published article and its supplementary information files or are available from corresponding authors on reasonable request.

## Abbreviations

BP: Biological processes
CC: Cellular components
DcS-ILP: *DcSto*-intron length polymorphism
*DcSto*: *Daucus carota Stowaway*
DH1: Carrot double haploid plant used for reference genome assembly
GO: Gene ontology
HTH: Helix-turn-helix
MF: Molecular functions
MITEs: Miniature inverted repeat transposable elements
MLE: *Mariner*-like elements
PIS: Parallel insertion sites
PrC: Proliferation coefficient
RdDM: RNA-directed methylation
SEA: Singular enrichment analysis
TEASV: TE-associated structural variation
TEs: Transposable elements
TIR: Terminal inverted-repeat
TSD: Target site duplication
TSS: Transcription start site
UIS: Unique insertion sites
UTR: Untranslated region
