# Supplementary material for: Stowaway miniature inverted repeat transposable elements are important agents driving recent genomic diversity in wild and cultivated carrot
Source: Mob DNA. 2019 Nov 27;10:47. doi: 10.1186/s13100-019-0190-3 (PMC6881990; doi:10.1186/s13100-019-0190-3)
Supplement: Supplementary file 2 — Additional file 2: Supplementary Figures and Tables: contains supplementary figures and tables referenced in the main manuscripts. [file 13100_2019_190_MOESM2_ESM.pdf]

Additional File 1. Supplementary Tables and Figures

Stowaway MITEs are important agents driving recent genomic diversity in wild and cultivated carrot

Alicja Macko-Podgórni[1*], Katarzyna Stelmach[1], Kornelia Kwolek[1], Dariusz Grzebelus[1*]

[1] Institute of Plant Biology and Biotechnology, Faculty of Biotechnology and Horticulture, University of Agriculture in Krakow, 31425 Krakow, Poland

* corresponding authors: Alicja Macko-Podgórni, a-mail: a.macko@urk.edu.pl; Dariusz Grzebelus, d.grzebelus@urk.edu.pl

Supplementary Table S1. List of accessions used for analysis and their characteristics.

No	ID	Taxonomy	Type	Origin	% Reads Mapped[c]	% Genome Coverage[d]	NCBI BioSample no	Specimen Voucher
1	DH1	*D. carota* subsp. *sativus*	double haploid	Rijk Zwaan	99.64	-	SAMN03216637	DH1
2	I1	*D. carota* subsp. *sativus*	inbred	USDA	97.42	94.13	SAMN03766317	B2566B 921-1 USDA
3	I2	*D. carota* subsp. *sativus*	inbred	USDA	97.52	94.63	SAMN03766318	B6274B 927-1 USDA
4	I3	*D. carota* subsp. *sativus*	inbred	USDA	96.73	94.19	SAMN03766319	B7262B 349-1 USDA
5	I4	*D. carota* subsp. *sativus*	inbred	USDA	94.64	96,10	SAMN03766320	B493B 920-1 USDA
6	C1	*D. carota* subsp. *sativus*	eastern cultivated	Afghanistan, Badakhshan	89.88	96.73	SAMN03766321	PI 211590
7	C2	*D. carota* subsp. *sativus*	eastern cultivated	China, Beijing	96.31	96.00	SAMN03766322	PI 652188
8	C3	*D. carota* subsp. *sativus*	eastern cultivated	Uzbekistan, Tashkent	96.40	95.79	SAMN03766323	PI 540422
9	C4	*D. carota* subsp. *sativus*	eastern cultivated	Afghanistan	96.83	94.76	SAMN03766324	PI 200876
10	C5	*D. carota* subsp. *sativus*	eastern cultivated	Syria, Damascus	95.59	95.96	SAMN03766325	PI 652336
11	C6	*D. carota* subsp. *sativus*	eastern cultivated	Turkey, Mugla	94.73	95.91	SAMN03766326	PI 652374
12	C7	*D. carota* subsp. *sativus*	western cultivated	Japan, Ibaraki	96.82	95.41	SAMN03766327	PI 652136
13	C8	*D. carota* subsp. *sativus*	western cultivated	Brazil, Rio Grande do Sul, Rio Grande	96.67	94.63	SAMN03766328	Brasilia Embrapa LOTE 39/06
14	C9	*D. carota* subsp. *sativus*	western cultivated	Netherlands	97.17	95.70	SAMN03766329	PI 261648
15	C10	*D. carota* subsp. *sativus*	western cultivated	USA, California	97.00	95.07	SAMN03766330	PI 643114
16	C11	*D. carota* subsp. *sativus*	western cultivated	Netherlands	96.31	94.87	SAMN03766331	PI 451755
17	C12	*D. carota* subsp. *sativus*	western cultivated	France	95.89	95.59	SAMN03766332	PI 264232
18	C13	*D. carota* subsp. *sativus*	western cultivated	USA, California	97.01	94.85	SAMN03766333	PI 632391
19	C14 [a]	*D. carota* subsp. *sativus*	western cultivated	Belgium	-	-	SAMN03766334	PI 187235
20	W1	*D. carota* subsp. *carota*	European wild	Portugal, Coimbra	94.54	95.33	SAMN03766342	PI 502244
21	W2	*D. carota* subsp. *carota*	European wild	Portugal, Beja	94.52	96.05	SAMN03766350	Ames 26408

22	W3	*D. carota* subsp. *carota*	European wild	France, Paris	96.12	93.92	SAMN03766336	PI 478861
23	W4	*D. carota* subsp. *carota*	Asian wild	China, Xinjiang	96.68	93.80	SAMN03766338	PI 478369
24	W5	*D. carota* subsp. *carota*	Asian wild	Uzbekistan, Gazelkent	96.79	95.25	SAMN03766335	Ames 27395
25	W6	*D. carota* subsp. *carota*	Asian wild	Turkey, Konya	95.47	96.82	SAMN03766343	PI 652393
26	W7	*D. carota* subsp. *carota*	Asian wild	Turkey, Izmir	96.60	93.98	SAMN03766337	PI 652358
27	W8	*D. carota* subsp. *carota*	Asian wild	Pakistan, Nomal	95.77	95.76	SAMN03766339	PI 274297
28	Ssp1	*D. carota* subsp. *gummifer*	European wild	Portugal, Faro	94.22	95.08	SAMN03766344	Ames 26381
29	Ssp2	*D. carota* subsp. *gummifer*	European wild	Portugal, Faro	94.45	95.21	SAMN03766345	Ames 26383
30	Ssp3 [b]	*D. carota* subsp. *gummifer*	European wild	France	94.95	94.75	SAMN03766351	Ames 31194
31	Ssp4	*D. carota* subsp. *gummifer*	European wild	France, Finistere, Le France, Conquet	95.53	96.00	SAMN03766341	PI 478883
32	Ssp5	*D. carota* subsp. *capillifolius*	European wild	Libya, Jefren	95.42	94.14	SAMN03766340	PI 279764

[a] accession not included in *in silico* analyses

[b] accession not included in the PCR verification

[c] results of bwa-mem read mapping to the reference DH1 genome

[d] percent of bases in the genome assembly with mapped reads, excluding Ns in the assembly, from [25]

Supplementary Table S2. Number of insertion sites attributed to each *DcSto* family, shared by one to 31 accessions.

No. of accessions sharing insertion sites	*DcSto1*	*DcSto2*	*DcSto3*	*DcSto4*	*DcSto5*	*DcSto6*	*DcSto7a*	*DcSto7b*	*DcSto7c*	*DcSto8*	*DcSto9*	*DcSto10*	*DcSto11*	*DcSto12*
1	1145	1739	489	153	916	2512	983	2284	143	637	140	184	72	1068
2	245	329	125	49	194	512	197	297	31	90	40	38	12	172
3	90	171	50	26	75	219	85	123	14	36	15	22	8	71
4	61	110	24	17	67	115	38	67	15	20	24	18	10	52
5	38	54	25	9	34	97	47	36	11	16	8	13	5	48
6	26	54	24	8	23	57	26	29	8	9	7	9	5	28
7	23	45	19	10	25	53	24	22	8	7	5	4	3	21
8	16	29	16	6	20	34	20	12	5	9	7	4	4	22
9	15	33	12	4	13	28	12	19	4	14	4	4	7	26
10	8	25	7	0	12	26	16	13	1	10	3	4	3	15
11	14	18	11	7	16	22	16	6	6	5	4	5	2	13
12	11	10	5	4	12	21	11	5	3	5	2	3	1	22
13	13	13	3	1	4	16	7	5	1	4	2	5	3	16
14	3	10	9	6	8	13	6	5	2	8	2	1	4	14
15	5	15	4	4	6	21	6	4	0	2	0	1	3	11
16	7	6	3	4	6	10	9	8	0	4	2	2	5	7
17	4	5	1	4	10	4	3	2	0	0	3	3	5	4
18	3	4	1	4	3	8	7	5	1	5	0	1	0	5
19	7	4	2	1	4	4	2	4	1	3	0	0	2	2
20	3	2	2	2	0	1	0	1	1	2	0	0	0	6
21	1	6	0	1	0	2	0	0	0	2	0	0	1	0
22	2	2	1	2	2	4	1	1	0	1	0	0	0	2
23	0	1	0	0	3	0	1	0	0	0	0	0	0	0
24	2	1	2	0	1	1	1	0	0	2	0	0	0	0
25	0	0	0	2	0	1	1	0	1	1	0	0	0	0
26	0	1	0	0	0	0	0	0	0	0	0	0	0	0
27	0	1	0	1	0	0	0	0	0	0	0	0	0	0
28	0	1	0	0	0	0	0	0	1	0	0	0	0	0
29	0	0	2	3	0	1	0	0	0	0	0	0	0	0
30	0	0	1	0	2	0	0	0	0	0	1	0	0	0
31	0	1	1	0	0	0	0	0	0	0	0	0	0	0

Supplementary Table S3. Insertion sites attributed to each *DcSto* family, shared by the cultivated carrots.

No. of accessions sharing insertion sites	*DcSto1*	*DcSto2*	*DcSto3*	*DcSto4*	*DcSto5*	*DcSto6*	*DcSto7a*	*DcSto7b*	*DcSto7b*	*DcSto8*	*DcSto9*	*DcSto10*	*DcSto11*	*DcSto12*
1	386	559	216	65	302	688	369	1622	70	85	69	70	25	285
2	127	212	87	29	127	273	139	205	21	37	25	21	7	119
3	77	128	30	13	65	137	67	94	13	22	21	28	7	60
4	46	73	25	14	50	77	34	68	7	18	16	13	10	38
5	34	57	21	6	26	79	33	24	12	15	8	12	6	44
6	25	46	25	12	25	54	25	33	9	10	6	8	3	33
7	20	38	20	11	19	47	23	17	5	7	6	3	5	22
8	14	28	10	7	19	32	15	10	3	10	5	8	7	21
9	13	22	11	2	17	26	15	14	4	12	4	1	4	21
10	14	20	6	3	13	26	23	13	2	7	3	8	5	21
11	14	18	10	6	9	22	12	8	4	8	1	6	3	11
12	10	17	4	8	12	18	11	8	2	6	1	3	1	19
13	7	12	3	6	10	13	9	6	0	7	2	2	4	13
14	6	7	7	2	8	11	5	6	3	4	2	2	3	4
15	5	11	2	4	9	9	5	5	0	3	1	1	3	9
16	6	8	3	2	2	4	7	3	2	3	0	0	1	6
17	2	3	1	3	3	5	2	2	1	4	0	0	1	4
18	0	5	5	3	1	2	1	0	1	1	1	0	0	2

Supplementary Table S4. Number of unique insertion sites (UIS) identified in the 31 accessions.

Accession	Genepool [a]	*DcSto1*	*DcSto2*	*DcSto3*	*DcSto4*	*DcSto5*	*DcSto6*	*DcSto7a*	*DcSto7b*	*DcSto7c*	*DcSto8*	*DcSto9*	*DcSto10*	*DcSto11*	*DcSto12*
DH1	WC	13	19	4	2	10	14	16	71	2	6	2	6	4	15
I1	WC	20	9	10	4	8	18	8	57	1	4	0	2	0	6
I2	WC	11	10	7	1	12	16	8	53	1	1	1	0	0	10
I3	WC	9	4	6	1	10	14	15	48	0	3	1	4	0	5
I4	WC	1	0	0	0	0	0	1	10	0	1	0	0	0	2
C1	EC	21	57	18	0	17	37	32	117	10	3	8	6	2	21
C2	EC	15	29	18	2	16	24	23	173	4	0	2	6	0	13
C3	EC	10	28	14	3	14	31	22	137	2	3	9	4	1	6
C4	EC	15	30	9	2	12	28	15	114	2	5	2	7	4	20
C5	EC	17	22	12	4	21	23	24	147	2	1	2	3	0	10
C6	EC	12	28	14	4	15	29	21	126	1	4	2	3	1	16
C7	WC	13	27	4	2	12	26	12	88	4	3	1	1	2	6
C8	WC	35	55	17	1	24	56	28	90	6	13	3	4	3	30
C9	WC	31	29	9	4	16	32	18	105	3	5	4	4	1	13
C10	WC	39	28	12	5	20	53	28	52	4	8	8	2	2	22
C11	WC	26	28	4	2	19	35	14	45	5	2	1	4	0	16
C12	WC	10	14	6	2	16	29	13	57	3	3	0	1	0	8
C13	WC	23	20	5	4	14	37	10	66	1	2	3	3	0	9
W1	EW	52	31	3	3	16	113	20	30	2	19	2	3	0	39
W2	EW	166	147	12	22	95	455	101	112	3	150	6	2	4	127
W3	EW	88	74	29	10	86	128	48	67	10	25	1	8	2	67
W4	AW	41	102	41	6	46	86	52	41	15	10	16	20	4	129
W5	AW	36	89	28	8	38	91	51	58	9	11	14	19	2	37
W6	AW	45	133	58	10	43	103	41	48	18	4	15	23	6	47
W7	AW	59	170	50	3	65	130	79	158	18	8	21	15	3	61
W8	AW	7	21	12	2	10	27	9	18	2	3	3	5	1	6
Ssp1	EW	37	54	5	5	28	187	23	30	0	68	1	2	4	43
Ssp2	EW	42	42	2	7	15	111	21	27	1	57	5	0	0	39
Ssp3	EW	143	139	21	22	86	397	76	73	1	143	4	2	7	117
Ssp4	EW	65	76	31	9	68	111	36	42	10	25	2	7	6	50
Ssp5	EW	43	224	28	3	64	71	118	24	3	47	1	18	13	78

[a] WC – western cultivated; EC – eastern cultivated; EW – European wild; AW – Asian wild

Supplementary Table S5. Significant GO enrichment of *DcSto*-associated genes with respect to BP[a] and MF[b].

MITE	Genic region	GO category	Description	GO number	p-value
All *DcSto*s	All insertions	BP	transcription, DNA-templated	GO:0006351	1.0e-08
		MF	transcription factor activity	GO:0001071<br>GO:0003700	3.7e-05
	Upstream	BP	regulation of transcription, DNA-templated	GO:0006355	1.6e-05
		MF	transcription factor activity	GO:0001071<br>GO:0003700	1.8e-05
	Downstream	BP	transcription, DNA-templated	GO:0006351	4.3e-10
		MF	transcription factor activity	GO:0001071<br>GO:0003700	8.0e-07
DcSto2	All insertions	BP	regulation of transcription, DNA-templated	GO:0006355	4.8e-05
	Downstream	BP	nucleic acid-templated transcription	GO:0097659	2.0e-04
		MF	transcription factor activity	GO:0001071<br>GO:0003700	0.028
DcSto5	All insertions	BP	regulation of transcription, DNA-templated	GO:0006355	2.5e-03
		MF	transcription factor activity	GO:0001071<br>GO:0003700	0.039
	Upstream	BP	regulation of transcription, DNA-templated	GO:0006355	1.6e-05
		MF	transcription factor activity	GO:0001071<br>GO:0003700	2.6e-03
	intron	BP	glucan and carbohydrate biosynthetic process	GO:0009250<br>GO:0016051	1.2e-05
		MF	glucosyltransferase activity	GO:0046527	3.8e-05
DcSto6	upstream	BP	nucleic acid-templated transcription	GO:0097659	1.3e-04
	intron	MF	hydrolase activity, acting on acid anhydrides	GO:0016818<br>GO:0016817<br>GO:0017111<br>GO:0016462	1.2e-03
DcSto7a	upstream	BP	regulation of gene expression	GO:0010468	6.8e-06
		MF	transcription factor activity	GO:0001071<br>GO:0003700	1.0e-05
DcSto7b	All insertions	BP	regulation of gene expression	GO:0010468	2.2e-08
		MF	transcription factor activity	GO:0001071<br>GO:0003700	1.5e-07
	upstream	BP	regulation of transcription, DNA-templated	GO:0006355	7.7e-04
	5'UTR	BP	nucleic acid-templated transcription	GO:0097659	5.8e-04
		MF	transcription factor activity	GO:0001071<br>GO:0003700	1.5e-05
	downstream	BP	regulation of transcription, DNA-templated	GO:0006355	1.8e-05
		MF	transcription factor activity	GO:0001071<br>GO:0003700	2.6e-04

[a] Biological process

[b] Molecular function

Supplementary Table S6. Relationship between PIS and genetic distance of consensus sequences representing *DcSto* families.

	DcSto1	*DcSto2*	*DcSto3*	*DcSto4*	*DcSto5*	*DcSto6*	*DcSto7a*	*DcSto7b*	*DcSto7c*	*DcSto8*	*DcSto9*	*DcSto10*	*DcSto11*	*DcSto12*
DcSto1		5	3	2	8	18	1	5	0	6	1	1	0	3
DcSto2	0.21		2	1	12	42	6	9	0	2	0	7	0	3
DcSto3	0.45	0.49		0	4	5	1	0	0	2	0	0	0	0
DcSto4	0.2	0.21	0.49		2	5	0	0	0	2	0	0	0	0
DcSto5	0.19	0.22	0.48	0.17		24	1	4	0	4	1	1	0	6
DcSto6	0.34	0.27	0.5	0.28	0.3		10	16	1	7	0	7	0	9
DcSto7a	0.52	0.48	0.51	0.44	0.52	0.46		8	1	0	1	1	0	5
DcSto7b	0.46	0.43	0.53	0.38	0.46	0.45	0.16		2	4	0	1	0	8
DcSto7c	0.46	0.49	0.46	0.47	0.52	0.5	0.17	0.26		0	0	0	0	1
DcSto8	0.33	0.41	0.53	0.31	0.32	0.43	0.51	0.51	0.47		0	2	0	0
DcSto9	0.45	0.47	0.46	0.42	0.46	0.43	0.36	0.4	0.41	0.43		0	1	0
DcSto10	0.47	0.49	0.45	0.48	0.47	0.49	0.4	0.42	0.39	0.47	0.36		0	0
DcSto11	0.45	0.46	0.27	0.45	0.48	0.48	0.41	0.44	0.35	0.51	0.38	0.36		0
DcSto12	0.47	0.51	0.41	0.43	0.5	0.52	0.44	0.45	0.48	0.45	0.38	0.48	0.4	

DcSto copies identified in parallel insertion sites (PIS) are presented as a upper triangle and genetic distances between the terminal 50 bp of their 5' and 3' TIRs as a lower triangle.

Supplementary Table S7. Presence of features characteristic for *Mariner*-like elements (according to [27]) in carrot *Dcmar*s.

Dcmar families	Genomic localisation in DH1	Length (bp)	TIRs (bp)	TIR sequence	DDD	HTH position	Mg^{2+} binding sites	Ca^{2+} binding sites
*Dcmar*1	CHR8: 25189375-25193731	4353	31	5'CTCCCTCCGTCCCTATTTATCTGTCCAMTTT3'	+	52	D193 D317 H322 E329 (DD39D)	D193 Q316 D317 N318 (DD39D)
*Dcmar*2	CHR2: 2329265 -2332914	3650	25	5'CTCCCTCYGTCCCRTKGGATWGTTA3'	+	114	D187 D310 (DD39D)	-
*Dcmar*3	CHR2: 2349703-2353394	3692	32	5'CTCCCTCCGTCCCTWAWTTCTTTTCYKGTTWT3'	+	52	D236 D360 (DD39D)	-
*Dcmar*4	CHR3: 43587365 - 43591144	3780	31	5'CTCCCTCCGTCCCAATTCTTTTGGCTGAKTT3'	+	52	D95 D219 (DD39D)	-
*Dcmar*5	CHR9: 10060874-10064362	3489	20	5'CTCCCTCTGGACCGATTWGT3'	+	-	D260 D384 N411 N420 D423 (DD39D)	D260 D384 D423 (DD39D)
*Dcmar*6	CHR8: 9590908 -9595847	4940	31	5'CTCCCTCTGTCCCAGTTTAWKAGTCGYTYTT3'	+	52	D124 D245 (DD39D)	-
*Dcmar*7	CHR5: 27436331 -27438744	2414	26	5'CTCCCTCYGTCCCKCTAWTATTGGCC3'	+	112	D156 D272 (DD39D)	-
*Dcmar*8	CHR7: 5627311 5630279	2969	28	5'CTCCCTCCGTCCCRCCAGGWTSTTTACA3'	+	-	D190 D313 (DD39D)	-
*Dcmar*9	CHR1: 9983042 -9984963	1922	24	5'CTCCCTCYGTCCCWWAAAGWTTGA3'	-	n/a	n/a	n/a
*Dcmar*10	CHR8: 10543661-10547402	3742	26	5'CTCCCTCCGTCCCATTTTATCTKGCT3'	-	n/a	n/a	n/a
*Dcmar*11	CHR2: 10935571-10939496	3926	31	5'CTCCCTCTGTCCCAGTTTAWKAGTCGYTYTT3'	-	n/a	n/a	n/a

Mismatched nucleotides within TIRs are underlined. The HTH position indicates distance between the last aa of the HTH motif and the first D of the DDD motif. Metal binding sites predicted for aspartic acids of DDD are underlined. Plus/minus is used to show presence/absence, n/a – sequence was not analyzed.

Supplementary Table S8. Number of genes associated with *DcSto* MITEs in the investigated genomes of *D. carota*.

ID	2 kb upstream	5'UTR	cds	intron	3'UTR	2 kb downstream	total
DH1	629	16	1	338	12	365	1361
I1	448	50	11	329	22	305	1165
I2	450	54	8	328	24	246	1110
I3	505	43	12	317	24	252	1153
I4	270	24	8	195	13	149	659
C1	594	62	5	353	39	292	1345
C2	537	76	5	344	26	293	1281
C3	533	71	8	369	46	287	1314
C4	521	65	4	326	30	255	1201
C5	541	72	11	306	33	279	1242
C6	494	64	11	312	39	300	1220
C7	523	57	7	329	32	275	1223
C8	495	52	6	397	30	302	1282
C9	583	70	12	429	35	361	1490
C10	489	68	14	380	43	326	1320
C11	428	53	12	315	30	278	1116
C12	552	61	12	395	36	268	1324
C13	554	55	13	349	25	313	1309
W1	119	22	0	105	15	76	337
W2	530	64	7	399	36	359	1395
W3	311	39	5	211	25	174	765
W4	495	75	9	356	35	281	1251
W5	531	71	8	374	32	304	1320
W6	484	64	15	326	36	266	1191
W7	474	76	9	302	34	288	1183
W8	302	39	2	199	16	152	710
Ssp1	189	28	4	196	16	130	563
Ssp2	154	21	4	169	11	114	473
Ssp3**	452	48	8	340	46	349	1243
Ssp4	217	35	3	186	14	139	594
Ssp5	247	39	2	190	17	153	648

Supplementary Table S9. List of primers used for DcS-ILP validation of *in silico* predictions.

Marker name	Chromosome	Forward primer sequences (5' --> 3')	Reverse primer sequences (5' --> 3')	Tm (°C)
DcS-ILP101	1	ATGGAGGAAGACTGGGGATT	GCCAAGCCCATAAATCTGAA	56
DcS-ILP105	1	GTAGAACACCCCCAGTCAGC	TGGGAAAGTTGCGAGATAGC	56
DcS-ILP110 [a]	1	CGTCTGGGAAGAAAGCTCAG	TTCTCCAGCAGCCACAGTAA	60
DcS-ILP111 [a]	1	TGGCGACTTGTGTAGGTGTT	GGGATTTCATGGTCAGAGGA	56
DcS-ILP119 [a]	1	TGTAGCAATGGCCTCAGGAT	CTCAGTTGCAGAGTTCAAGCA	56
DcS-ILP201 [a]	2	TGCTTCTATCTGGATGCTCTTG	TTCGAAGGACAATCTGAGGA	59
DcS-ILP202 [a]	2	TCCTGAACCAGTCATTGCAC	CGGAGCATTAACGACATTCA	56
DcS-ILP207 [a]	2	TCAGCTGTTTTGACCAGCAT	GAACATCTTCCATCCGCTTG	56
DcS-ILP209 [a]	2	TCGTCGACATGCTTCCTACA	GGCACATTAAAAGGCATGGA	56
DcS-ILP214 [a]	2	CGTGTGCATTTTAGCTGCTG	CTCGAGGATATCAGAAACGAGA	56
DcS-ILP222 [a]	2	GCGAATCCTTTAGCAGGTTG	ACAGCCACAGTTTGCTACATT	56
DcS-ILP224 [a]	2	TGGAGAATTGCAGCTACGAC	GGACCCTGAACGCAACTATC	56
DcS-ILP226 [a]	2	TAGAGTCGGTCCGTTGGACT	CTGGCACCACATTATCCTCA	56
DcS-ILP229 [a]	2	GCATAGTCATCAAGGCATGG	CATGATCAGGGGCACATCT	56
DcS-ILP230 [a]	2	GTCCAGGGACCTCCTGTGT	GGCTTCTAAACTGGTCAAGGAA	56
DcS-ILP309	3	AAATGTTCGCTTTCAGCATGT	GCCATTTAATCCGATCCTTG	56
DcS-ILP313 [a]	3	CGGAACAAGCTCAAATAGGG	TGTGATGCAGTGGACACTTATG	56
DcS-ILP315	3	ACGGCCCTACTTACCATGC	AAGAATAGCAACCGACACCAA	56
DcS-ILP317 [a]	3	TTCTCTCAGGAAGATACGATGC	GGGGTGATTTCACTGCTGAT	56
DcS-ILP-318	3	CTAGCAGCAACAGCCATCTC	AAAAGTCTCGGTTGGGAAGA	58
DcS-ILP319 [a]	3	CCCAACAGACAAAAGTGCTG	GCCATCCTTCTTCCTTTCCT	56
DcS-ILP407 [a]	4	CTTCCACATTGGCAATTCCT	CCTGACATTTAGGGCTGATTTT	56
DcS-ILP409 [a]	4	CAATCTCGCCATTTTCTGGT	CGAGAAGCTCAACTGGAACC	56
DcS-ILP412	4	CGAGCTGCTGACACTACTGC	GTCCATCACTTCCGTCACCT	56
DcS-ILP417 [a]	4	GGCTGAAGTTGTGTGCAAGA	AGTTTATCCCCTGCGTTGTG	56
DcS-ILP425 [a]	4	GAAAAGGAGCAAGGTCATCG	TGGAATCACTTCGTGCTGAG	58
DcS-ILP511 [a]	5	TGATCCTCAACGGATCAAGT	TCAAAGCTTCGACCCATACC	56
DcS-ILP515	5	TTGGGGCTATGCAGAGAGTT	CCACGGAAACATGGATAATCA	56
DcS-ILP522	5	TCCTTTAAAATAGAGGGCGTTG	GCATGTCGTCAGGTTGATTTT	56
DcS-ILP524 [a]	5	GAACAGGAACCGAATCATCAA	GAAGCCCTTTGACGTCTTTCT	56
DcS-ILP612 [a]	6	CTGGGCAATCCTTGAGATGT	TCGCACTAGTGAGGTTGTGC	56
DcS-ILP613	6	TGGTTATGGACTTGCAAAGG	CATGAGTCCTCCACCAACCT	56
DcS-ILP616 [a]	6	GACGGTGATCGACATCTTGA	GCTTTCGTTCGTCGAATCTC	56
DcS-ILP619 [a]	6	AAAGCACTTGCTTCCTTCCA	GCTGGTAACCAAGGTTTTGG	56
DcS-ILP621 [a]	6	TGGATCGTTACCCAACTCAA	GTAAAGGGGCAGCTGTTAGG	58
DcS-ILP701 [a]	7	CAAGACGCTCTGGTGAAGTT	TCCAGGCAGGAATATTCAGA	56
DcS-ILP710	7	GAATTGGCATGGTTCAGATG	CATCACGAGCTGCTTGTTCT	56
DcS-ILP711	7	GGCTGCGGAATACTCGTTAT	GAAGCCTCCTCAAACTCTGG	56
DcS-ILP717 [a]	7	TGAGTTACACCGGCATCTTC	TGAGGACAGTTCTGCACATG	56
DcS-PIS309	3	TAGCCTGTCCCTTTTGCATT	TCAATTTTGGTGGCATGGTA	56
DcS-PIS410	4	TGGTCATGTTGCTTTGCTTC	AGAGGCCATTCGGCTTGTAT	56
DcS-PIS411	4	TTCTGCCCCTTTCTTTTGTG	CATGCCAACTCCTTTTGCTT	56

DcS-PIS820	8	CAGCTTCACATTGTACTTTTTGC	TCTTGCCAATGCTTCTTCCT	56
DcS-PIS922	9	ACAGCAAATTCGAGCTGCTT	GCTGCCCACTCATCACACTA	56
DcS-PIS923	9	GTTGCAGAGGATTTCCAAGC	CCTTGTTGTTTGCCTTGACA	56
DcS-PISH301	3	TTACCCTGGCTACGCTTCCC	AATTCACGAGGACGCAACACC	
DcS-PISH402	4	GTGAAGAGTGAAATGCTTGCT	GCTCAAACAGGTCAAAACACATC	
DcS-PISH503	5	ATGTGTATGTCAGTAGCATGTGT	AGACAAATTACGCAGATGGACAA	
DcS-PISH604	6	CCCACACACTCTCCCGAAAT	GCCGTGTAGTACCAAACCCA	
DcS-PISH708	8	AATGCTAATCTAACTCTTTGCCTG	CGGTGCTCAAGGGTTTAAGTTTT	

[a] insertion sites producing unambiguous amplicons, excluding PIS

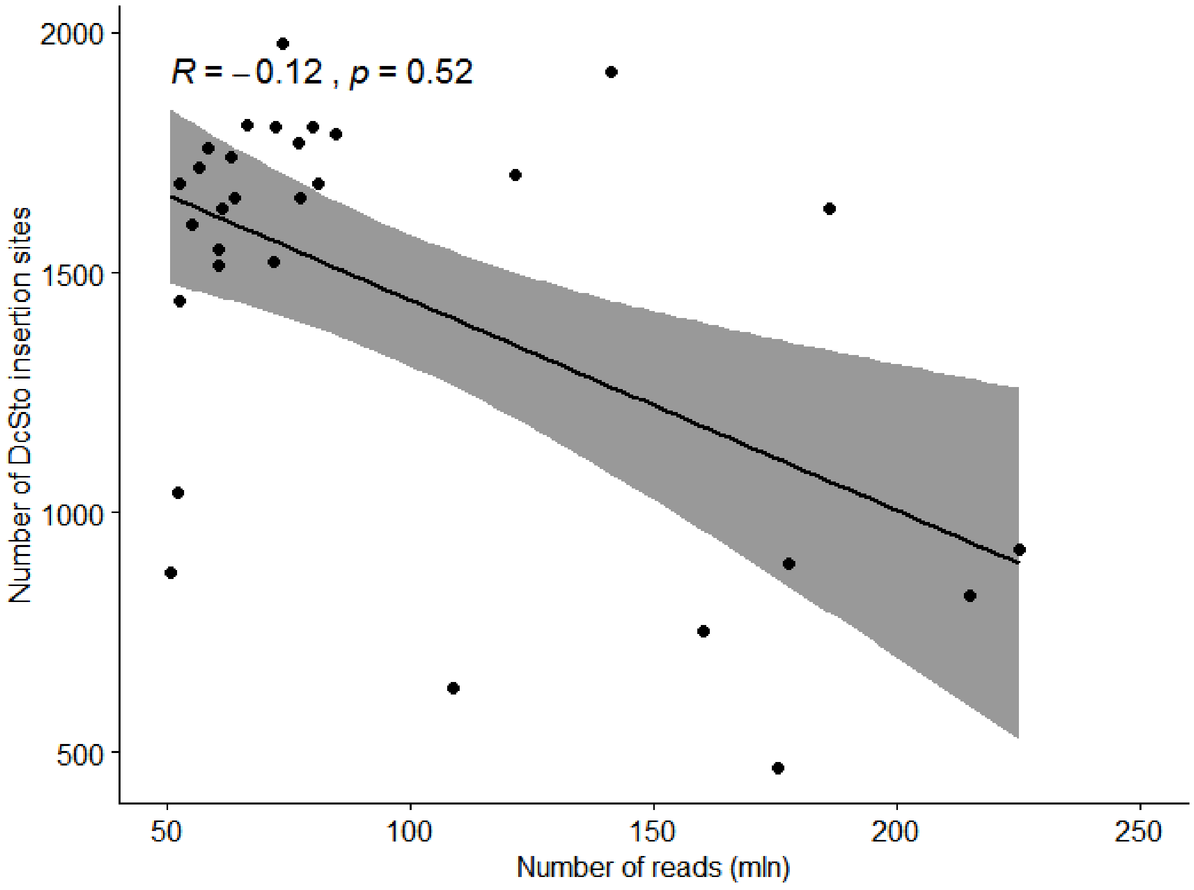

Supplementary Fig.S1. Relationship between the number of reads and the number of identified *DcSto* insertion sites. Regression line is shown in black along with gray-colored 0.05 confidence interval.

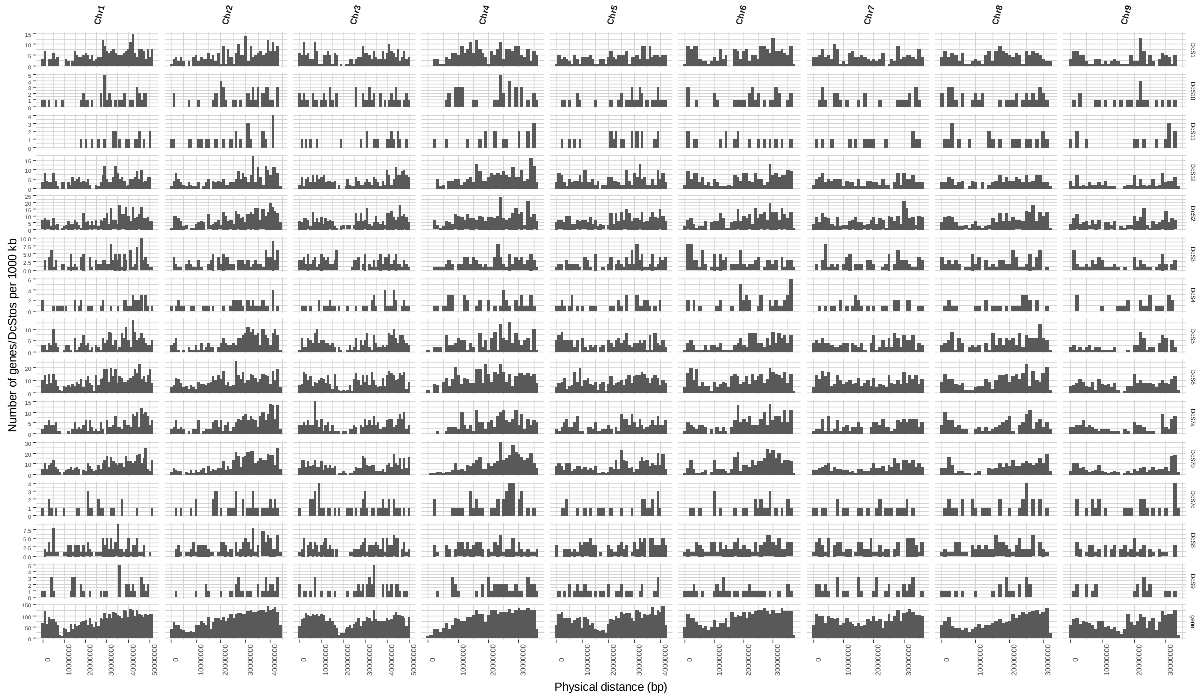

Supplementary Fig.S2. Distribution of *DcSto* copies and genes along carrot chromosomes. Scales of the Y axes were adjusted according to the number of copies per family or genes.

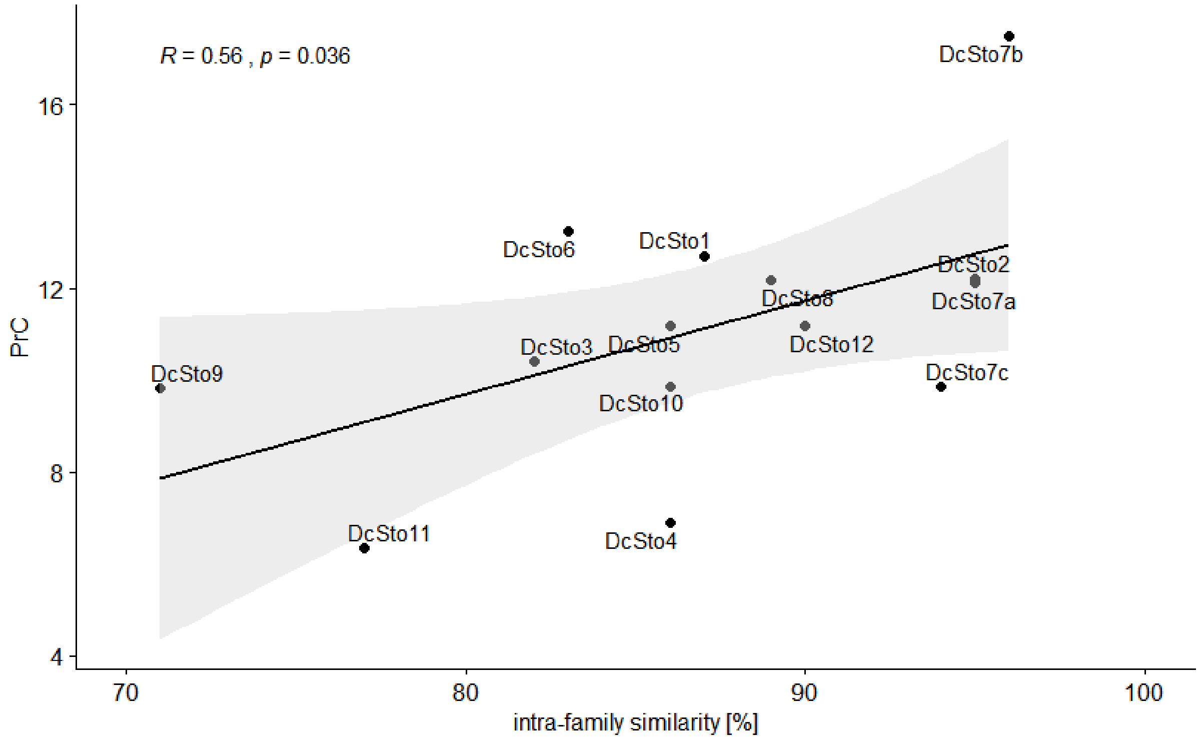

Supplementary Fig.S3. Correlation between proliferation coefficient (PrC) and intra-family similarity of 14 *DcSto* families in *D. carota*

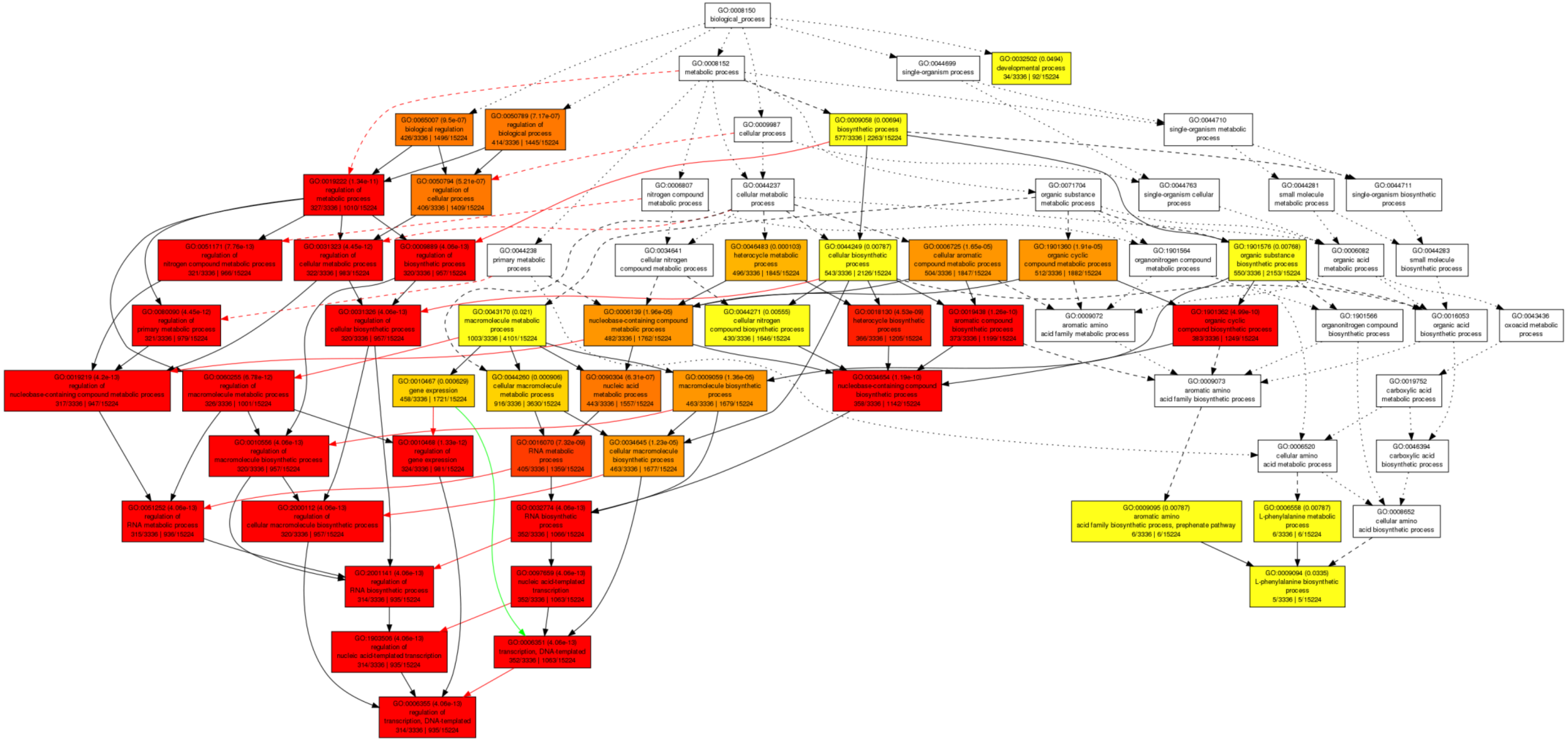

Supplementary Fig.S4. Singular enrichment analysis (SEA) of the *DcSto*-associated genes using AgriGO with respect to biological processes.

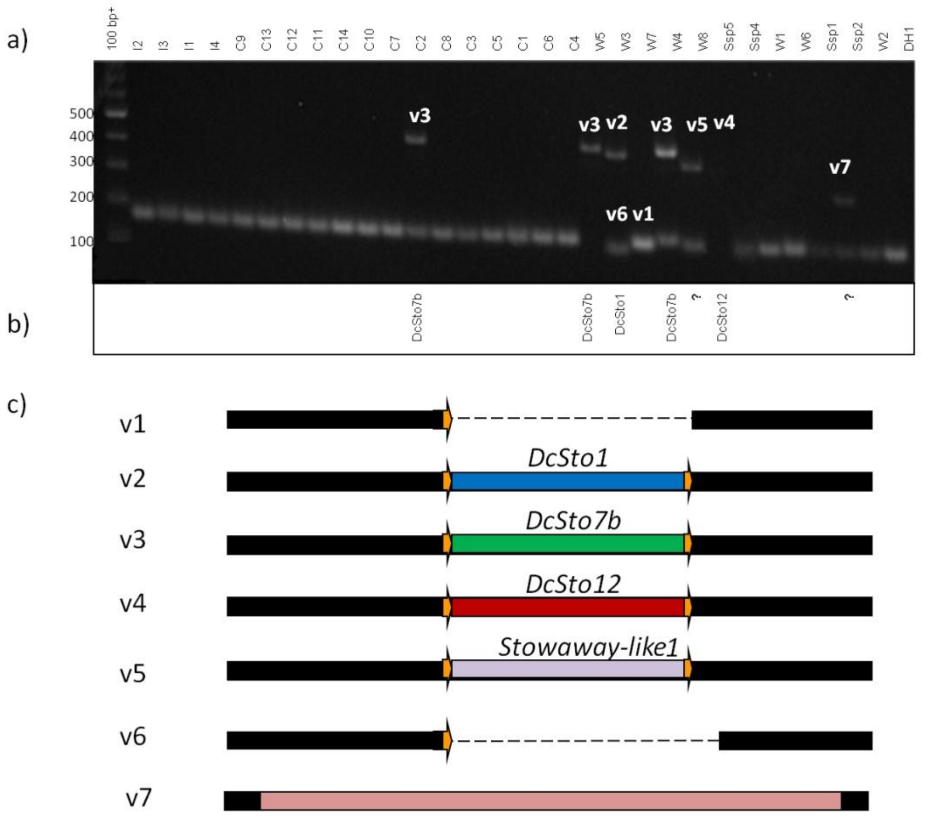

Supplementary Fig.S5. Verification of multiple parallel insertions (PIS) in the DcS-MIS410 site. a amplification profiles with variants (v1 to v7) labeled according to **c**; **b** insertions identified using RelocaTE; **c** a schematic representation of all insertion variants. TS (target site) is represented by orange arrow.

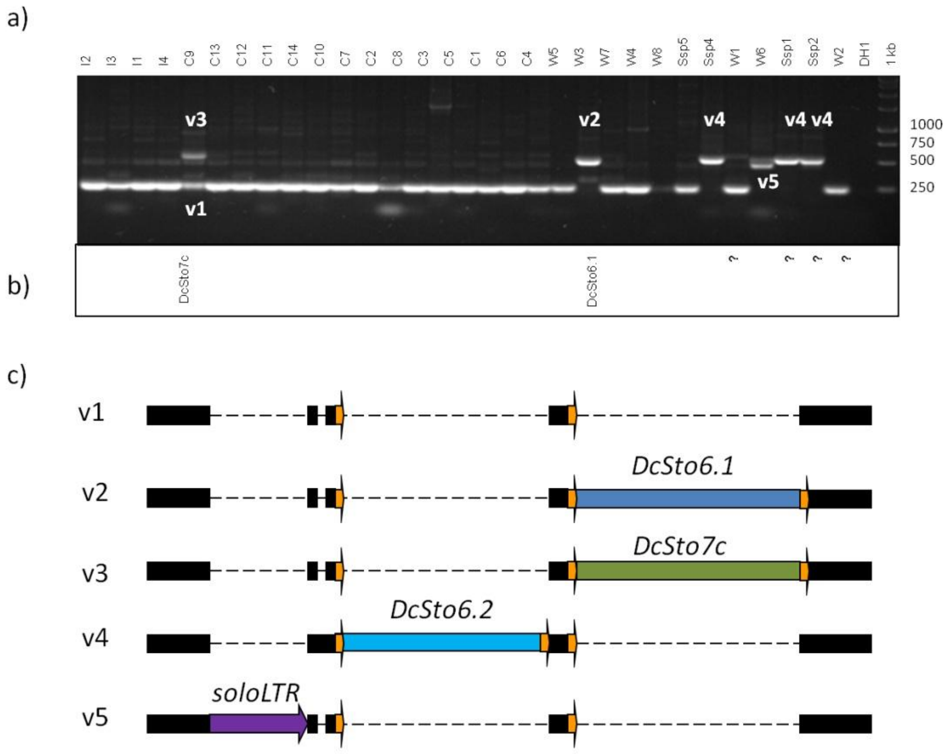

Supplementary Fig.S6. Verification of multiple parallel insertions (PIS) in the DcS-MIS922 site. **a** PCR amplification profiles with variants (v1 to v5) labeled according to **c**; **b** insertions identified using RelocaTE; **c** a schematic representation of all insertion variants. TS (target site) is represented by orange arrow.

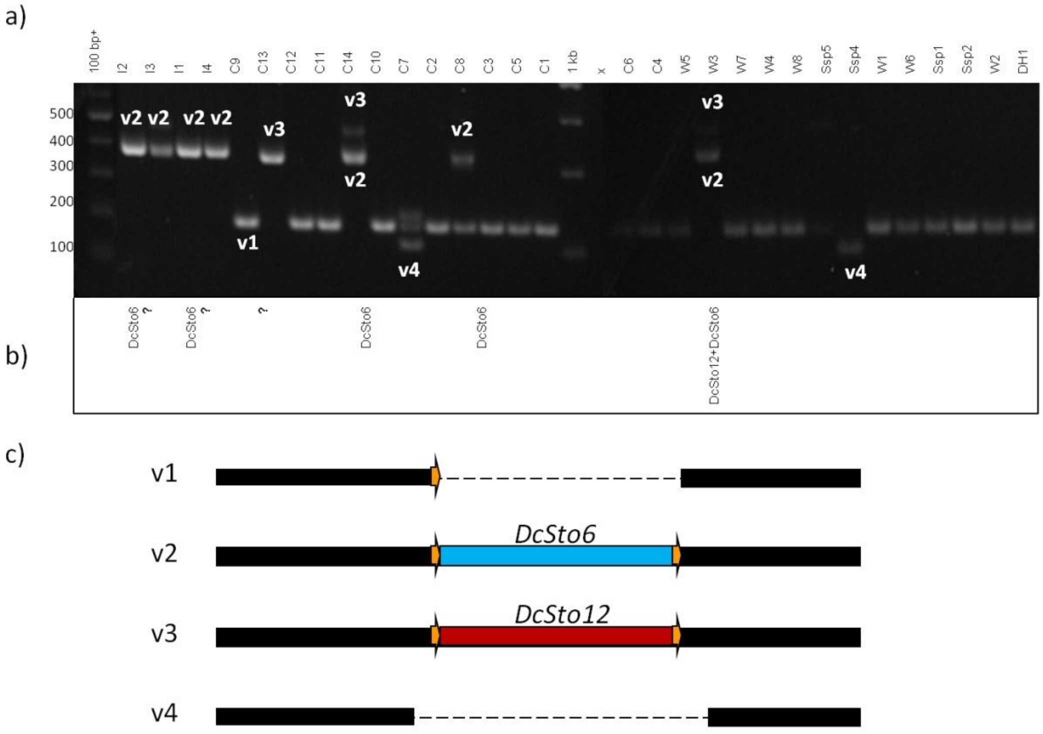

Supplementary Fig.S7. Verification of multiple parallel insertions (PIS) in the DcS-MIS411 site. **a** PCR amplification profiles with variants (v1 to v4) labeled according to **c**; **b** insertions identified using RelocaTE; **c** a schematic representation of all insertion variants. TS (target site) is represented by orange arrow.

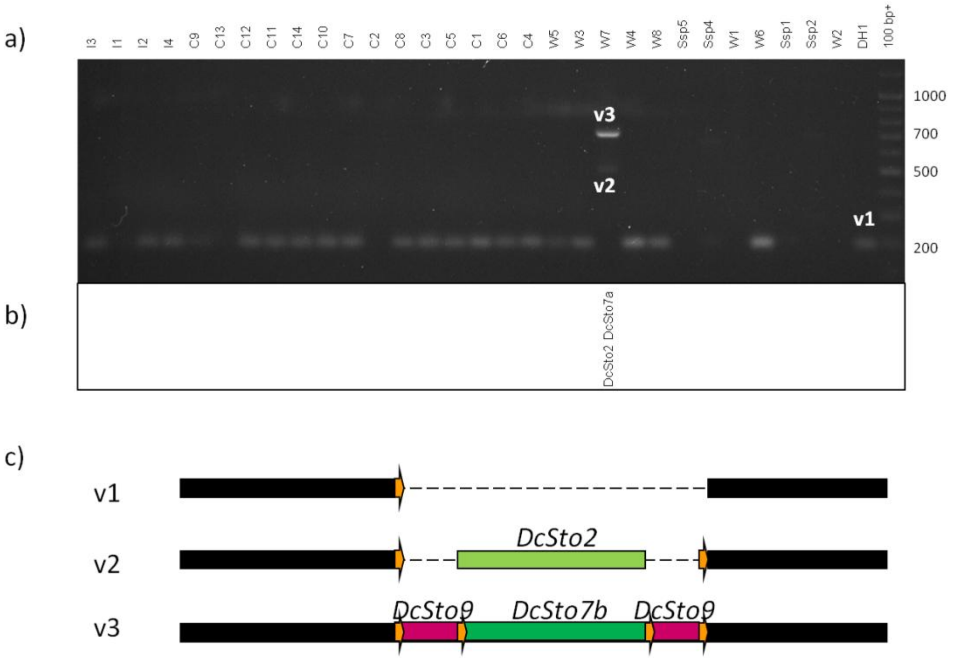

Supplementary Fig.S8. Verification of multiple parallel insertions (PIS) in the DcS-PISH301 site. **a** PCR amplification profiles with variants (v1 to v3) labeled according to **c**; **b** insertions identified using RelocaTE; **c** a schematic representation of all insertion variants. TS (target site) is represented by orange arrow.

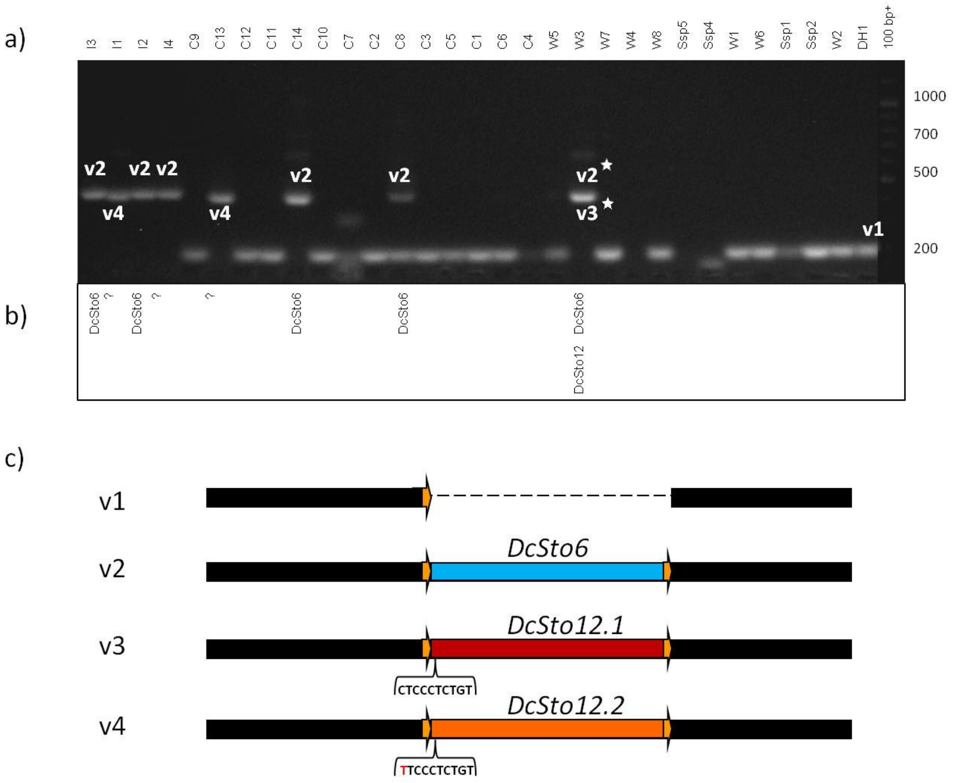

Supplementary Fig.S9. Verification of multiple parallel insertions (PIS) in the DcS-PISH402 site. **a** PCR amplification profiles with variants (v1 to v6) labeled according to **c**; **b** insertions identified using RelocaTE; **c** a schematic representation of all insertion variants. TS (target site) is represented by orange arrow. Heterozygous allelic variants of similar size, determined by cloning and sequencing, are labeled with white asterisks.

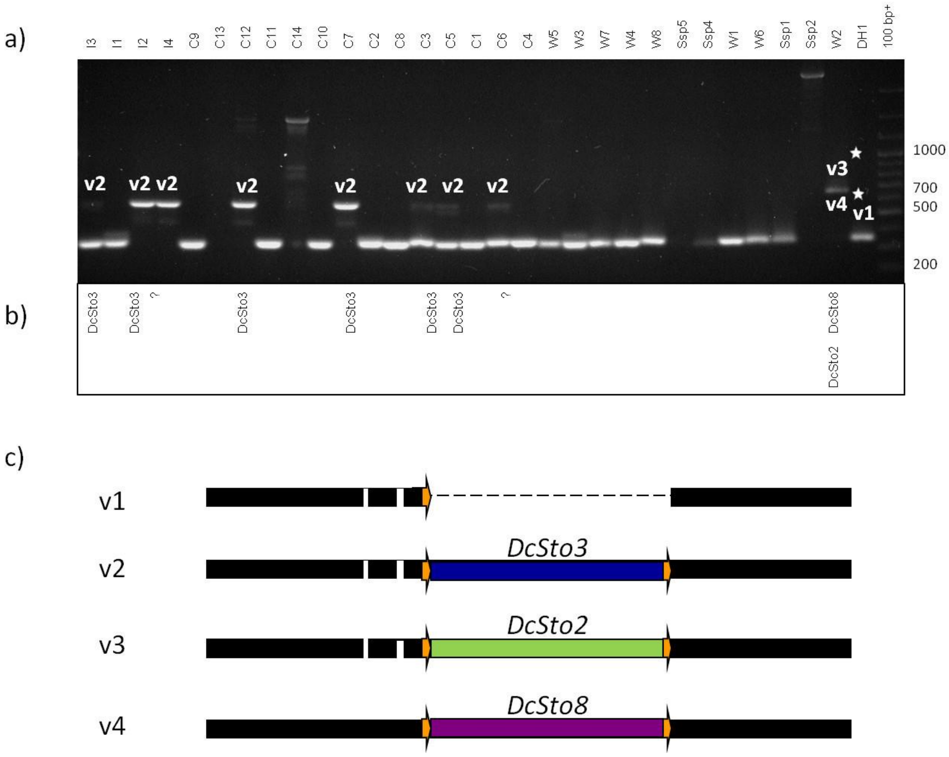

Supplementary Fig.S10. Verification of multiple parallel insertions (PIS) in the DcS-PISH503 site. **a** PCR amplification profiles with variants (v1 to v6) labeled according to **c**; **b** insertions identified using RelocaTE; **c** a schematic representation of all insertion variants. TS (target site) is represented by orange arrow. Heterozygous allelic variants of similar size, determined by cloning and sequencing, are labeled with white asterisks.

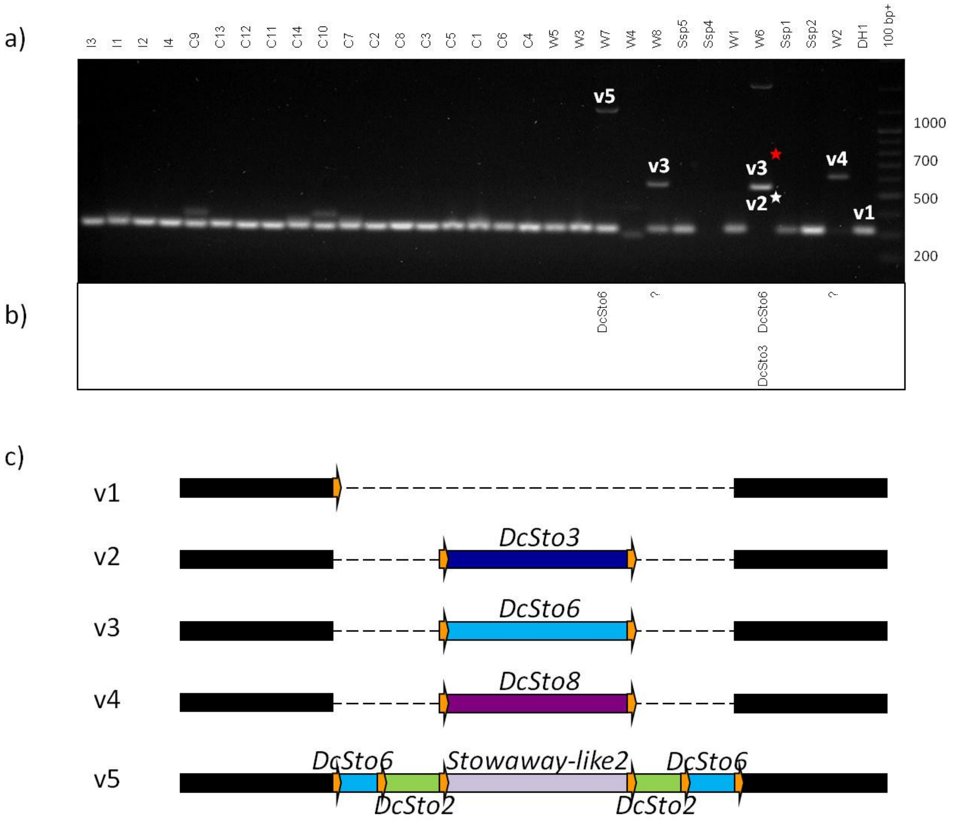

Supplementary Fig.S11: Verification of multiple parallel insertions (PIS) in the DcS-PISH604 site. **a** PCR amplification profiles with variants (v1 to v6) labeled according to **c**; **b** insertions identified using RelocaTE; **c** a schematic representation of all insertion variants. TS (target site) is represented by orange arrow. Heterozygous allelic variants of similar size are labeled with white and red asterisks for variant confirmed and not confirmed by cloning and sequencing.

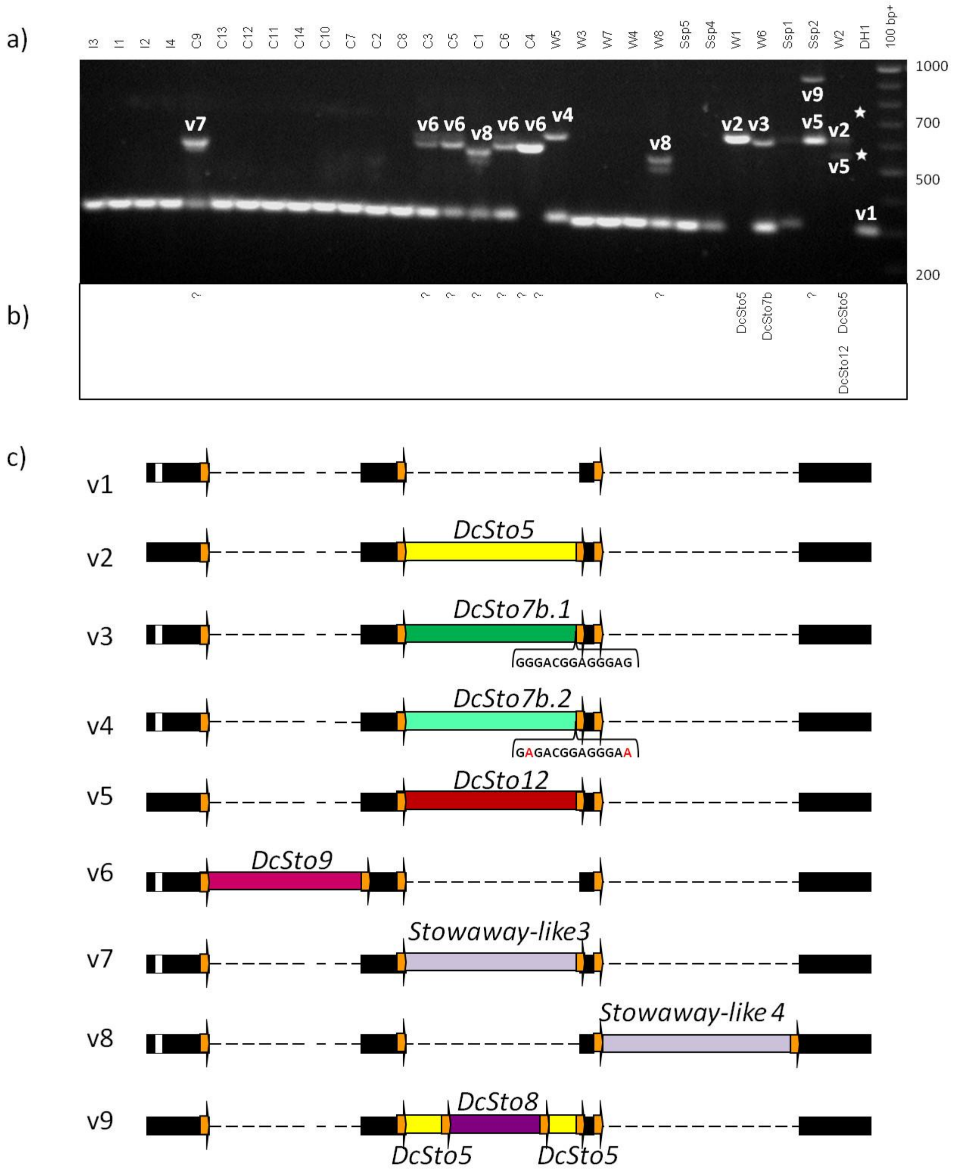

Supplementary Fig.S12. Verification of multiple parallel insertions (PIS) in the DcS-PISH708 site. **a** PCR amplification profiles with variants (v1 to v6) labeled according to **c**; **b** insertions identified using RelocaTE; **c** a schematic representation of all insertion variants. TS (target site) is represented by orange arrow. Heterozygous allelic variants of similar size, determined by cloning and sequencing, are labeled with white asterisks.

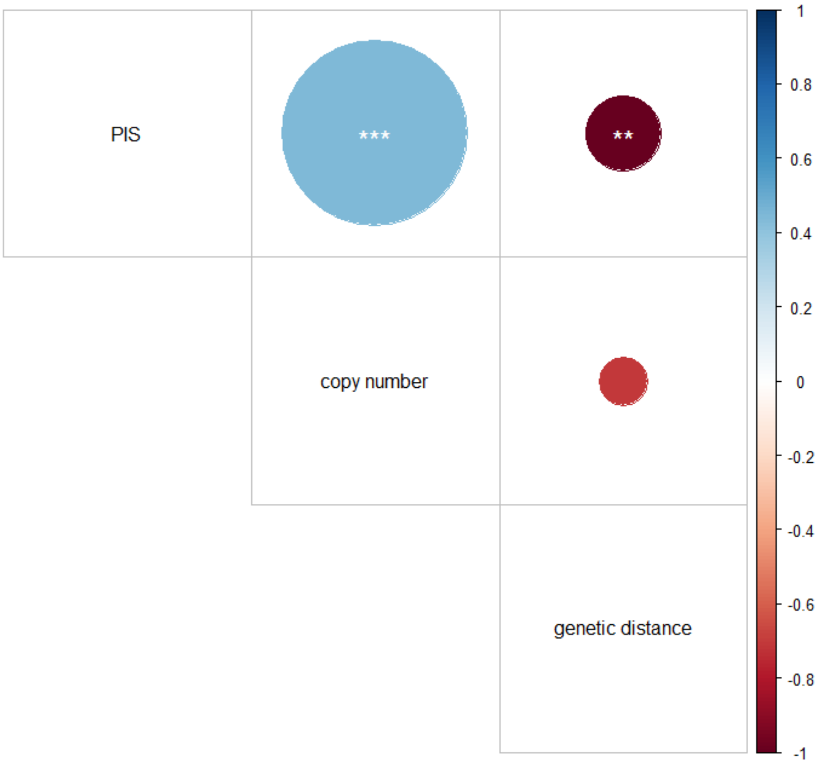

Supplementary Fig.S13. Correlation between each two *DcSto* families PIS, copy number and genetic distance.

Positive correlation of the cumulative copy number for families having copies inserted into the same position (PIS) and the number of their occurrences in PIS (blue) and negative correlation of genetic distances between each pair of *DcSto* consensus sequences and the number of their common occurrences in PIS (red). Significance levels: *** p=0.001, ** p=0.01.

a)

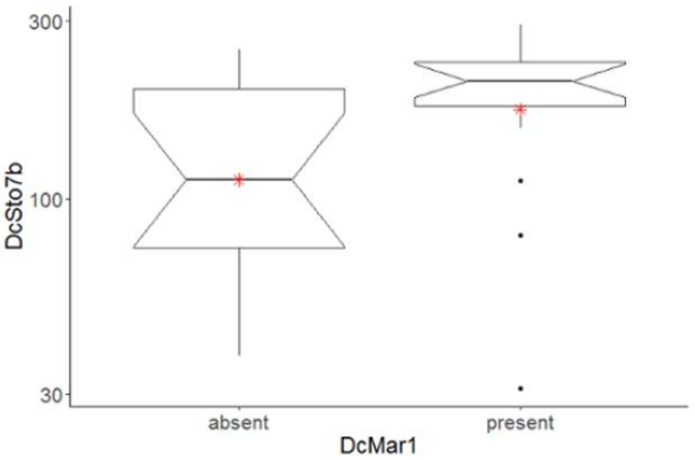

DcSto	p-value	mean DcMar1-	mean DcMar1+
DcSto1	0.8274	130.64	134.23
DcSto2	0.2029	194.57	227.29
DcSto3	0.3956	73.00	83.94
DcSto4	0.3163	42.57	48.35
DcSto5	0.2854	115.64	130.88
DcSto6	0.8808	277.57	272.00
DcSto7a	0.0582	104.64	137.35
DcSto7b	0.0290	132.57	191.64
DcSto7c	0.3045	23.14	28.29
DcSto8	0.9976	70.43	70.47
DcSto9	0.3258	24.21	29.41
DcSto10	0.3066	26.93	33.41
DcSto11	0.0338	20.14	28.06
DcSto12	0.3012	132.21	149.82

b)

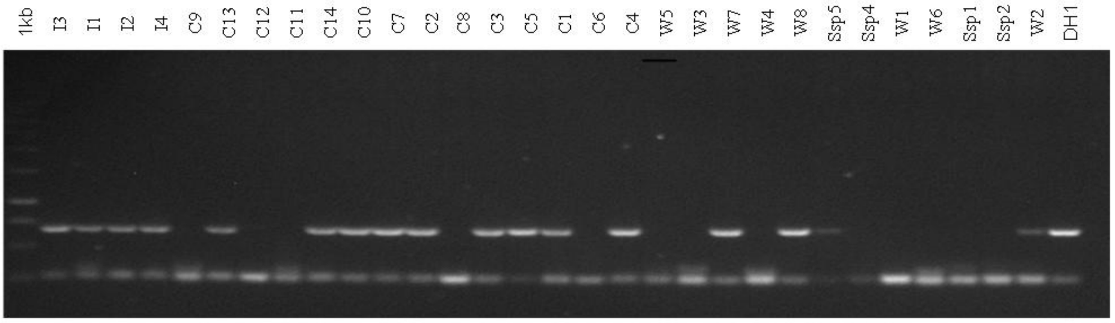

c)

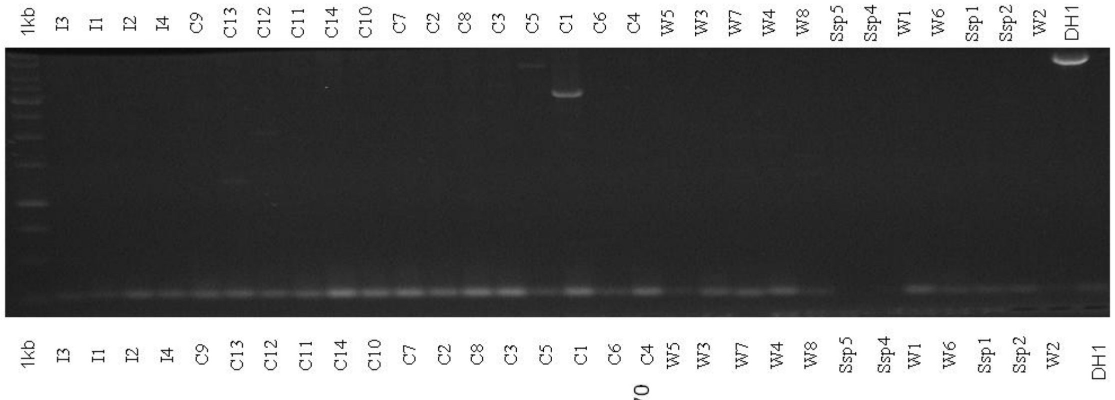

d)

chr2:207988 3' UTR
repetitive region
repetitive region
na
not found
repetitive region
not found
not found
repetitive region
chr6:27396182 upstream of gene
chr6:30012964 upstream of gene
chr4:17909803 upstream of gene
not found
repetitive region
chr6:34131158 upstream of gene
repetitive region
not found
chr2:42125926,chr6:8690970 inergenic
not found
not found
repetitive region
not found
chr2:39634672 intron
chr3:28483478 upstream of gene
not found
not found
not found
not found
not found
not found
chr8:25189375 intron

Supplementary Fig.S14. Occurrence of *Dcmar1* in genomes of 31 cultivated and wild carrots.

a Box plot showing a relationship between the presence of *DcMar*1 and log10 *DcSto*7b copy number, p-values of independent 2-group t-test showing relationships between the presence of *DcMar*1 and copy numbers of all *DcSto* families. Mean is shown as the red asterisk and median is indicated by the black line. **b** PCR assay determining presence or absence of *Dcmar*1 in genomes of *D. carota* accessions, **c** amplification of the *Dcmar*1 insertion site in DH1, indicating that *Dcmar*1 in other accessions resides elsewhere in their genomes, and **d** results of *in silico* identification of *Dcmar*1 positions in other genomes. Primers anchored in the DDD domain were used in **b**, while one DDD-anchored primer in combination with a primer anchored in the region flanking the *Dcmar*1 insertion in DH1 were used in **c**. The longer fragment is the DDD domain in **b** and the 'occupied' insertion site in **c**, while the shorter fragment is a positive control of amplification.
